# Parent perspectives on services available to autistic children in Canada: A mixed methods survey study

**DOI:** 10.1371/journal.pone.0353678

**Published:** 2026-08-20

**Authors:** Isabelle Caven, Yani Hamdani, Yona Lunsky, Courtney Weaver, Melanie Penner

**Affiliations:** 1 Institute of Medical Science, University of Toronto, Canada; 2 Bloorview Research Institute, Holland Bloorview Kids Rehabilitation Hospital, Canada; 3 Department of Occupational Science and Occupational Therapy, University of Toronto, Canada; 4 Rehabilitation Sciences Institute, University of Toronto, Canada; 5 Azrieli Adult Neurodevelopmental Centre, Centre for Addiction and Mental Health, Canada; 6 Department of Psychiatry, Temerty Faculty of Medicine, University of Toronto, Canada; 7 Autistic Advisor, Canada; 8 Department of Paediatrics, University of Toronto, Canada; Hacettepe University: Hacettepe Universitesi, TÜRKIYE

## Abstract

**Objectives:**

Given the differing opinions among interest holders on services that are commonly accessed by autistic children, exploration of parent perspectives is needed to contribute to this knowledge base. Our objective was to explore perspectives of parents of autistic children on select services accessed by autistic children in Canada.

**Methods:**

Using a mixed methods survey, the perspectives of parents of autistic children living in Canada on occupational therapy (OT), speech-language pathology (SLP) services, and applied behaviour analysis (ABA) based therapies were sought. The Theoretical Framework of Acceptability (TFA) and its constructs were used to develop the survey and served as a framework for qualitative content analysis.

**Results:**

Eighty-seven parents completed the survey. Acceptability scores were higher for OT and SLP compared to ABA (F (2, 239) = 43.42, p < 0.01). Related to the TFA construct of *Ethicality*, parent respondents shared concerns about potential harms of negative therapy experiences, emphasized the value of neurodiversity-affirming services and held mixed views on behavioural approaches. Under the construct of *Perceived Effectiveness*, acceptability was contingent on parents attributing improvements to the therapy and therapist fit. For the construct of *Self-Efficacy*, respondents reported the importance of therapist engagement with parents.

**Conclusions:**

Across OT, SLP, and ABA, *Ethicality*, *Perceived Effectiveness*, and *Self-Efficacy* informed the perceived acceptability of autism services. This work highlights that many parents are concerned with promoting a positive autistic identity for their children, including through therapies. Future work should seek to develop shared definitions and goals for therapy between clinicians, parents/care partners, and autistic people.

## Introduction

Autistic children, their families, and healthcare providers collaborate to determine what services can help children meet their goals and needs [1], which may include speech-language pathology (SLP), occupational therapy (OT), and applied behaviour analysis (ABA)-based services. Various terminology is used to describe services that may be accessed by autistic children. Use of the term ‘intervention’ is not supported by autistic communities, as autism is not an illness nor disease and implications relating to ‘curing’ autism are viewed as offensive. However, many studies that describe early services accessed by autistic children use the term ‘intervention’. Where these studies are described, the term ‘intervention’ is used to maintain consistency. For the purposes of this paper, the terms ‘services’ and ‘therapies’ are used to describe OT, SLP and ABA. Increasingly, there is public and documented debate about the acceptability of autism services, informed by the perspectives of both autistic people and parents/care partners of autistic children [1]. The voices of autistic adults were not traditionally sought out through research; however a growing number of calls to action [2,3] and frameworks [4,5] provide researchers with the tools to engage those with lived experience. This dialogue is connected to the mounting emphasis on neurodiversity, defined as human diversity in cognition and brain function [6], and stressing that there is no ‘right’ style of brain functioning [7]. In clinical practice, there are many ways to incorporate neurodiversity-affirming principles, which broadly focus on respecting autistic ways of knowing and being. Some examples that have been put forward include integrating multiple forms of communication, centering child enjoyment and meaningfulness of activities, respecting self-stimulatory behaviours, and identifying potential environmental triggers [8].

More recent research is exploring the perspectives of autistic people on various autism services. A thematic analysis of a social media group (*n* = 156 posts) and 10 podcasts found that autistic adults supported therapies that affirmed autistic identities and concentrated on environmental accommodations, self-advocacy, and autonomy [9]. A new survey study similarly identified high autistic acceptability of educational goals that promote individual autonomy (e.g., self-help skills, navigating routines) [10]. In contrast, a qualitative study interviewed eight autistic people about social communication interventions they had received [11]. One of the identified themes was a belief that the interventions were “ineffective and unnecessary”, as some respondents saw no tangible outcomes from therapy and others could not recall specific therapeutic goals [11]. In a qualitative study of seven autistic adults aged 18–27 years who had received ABA in childhood, respondents identified some benefits from ABA (e.g., learning about personal space and communication skills). However, most respondents described their experiences as abusive or traumatic and attributed negative long-term consequences to ABA participation (e.g., mental illness and falling behind academically) [12].

While autistic perspectives are critical to inform autism services, parental perspectives are also essential as parents carry significant responsibility in selecting these services, especially for younger children. However, there is limited research on parents’ perspectives on ABA, OT, and SLP. A recent survey explored therapy goals for young autistic children from the perspectives of parents, as well as autistic adults and professionals. Across all three groups, reducing or replacing behaviours that result in autistic children harming themselves or others (e.g., head banging, biting, hitting) was identified as a high priority [13]. Studies on parent perspectives often focus on reported satisfaction with various services without further exploration of the specific aspects of services that shaped their perspectives. For example, an Irish survey found high parental satisfaction with early intensive behavioural intervention [14]. By contrast, an Australian qualitative study of 20 parents found unmet expectations related to goal setting, treatment approaches, and the rationale provided for SLP services [15].

Social validity has long been used as a framework for assessing autism interventions, which explores the extent to which the goals, procedures and outcomes of interventions are perceived as acceptable and valuable by key parties [16]. However, social validity assessment has been criticized for its lack of rigor [17]. Outside of social validity, existing research has not explored parents’ perspectives on autism services through the lens of a theoretical framework, with one exception, which used critical theory within a phenomenological framework [12]. Acceptability, defined as “the extent to which the person delivering or receiving the healthcare intervention considers it to be appropriate, based on anticipated or experiential cognitive or emotional responses to the intervention” [18] is a well-described construct in the health services literature and, to date, has not been used to explore therapies accessed by autistic children. The Theoretical Framework of Acceptability (TFA) provides a comprehensive structure for evaluating perceived acceptability [18]. The TFA consists of seven constructs – *Affective Attitude*, *Intervention Coherence*, *Perceived Effectiveness*, *Self-efficacy*, *Burden*, *Opportunity Costs*, and *Ethicality*. The constructs included in this framework provide further breadth than offered by social validity. This framework promotes exploration of how goals and methods align within individual value systems, beyond the extent to which these goals and methods are ‘socially’ appropriate. Exploring the *Burden*, *Opportunity* *Costs* and *Perceived* *Effectiveness* is especially relevant to services accessed by autistic children and their families, which can be costly and time intensive. Further, inclusion of multiple modalities (OT, SLP and ABA) provides an opportunity to compare acceptable features of each approach.

Three therapies were chosen for this survey as an Ontario-based survey found these services to be the most popular amongst parents (80% would choose ABA for their child, 58% would choose SLP, 58% would choose OT) [19]. Broadly speaking, the aim of ABA is to teach different skills by encouraging, or reinforcing, behaviours required for that skill [20]. ABA is inclusive of early intensive behavioural intervention (EIBI), discrete trial training (DTT) [21], and newer models that integrate a developmental approach, such as pivotal response training and the Early Start Denver Model (EDSM) [22]. According to Speech-Language & Audiology Canada, SLPs support individuals with a variety of issues, including speech delays, language delays, swallowing and feeding disorders, cognitive-communication issues, and literacy skills [23]. For autistic children, SLPs support developing interpersonal communication strategies that are uniquely suited to a child, such as augmentative and alternative communication [24]. According to the Canadian Association of Occupational Therapists, OT “promotes health, well-being, and quality of life by supporting access to, initiation of, and sustained participation in the things that clients want and need to do in their daily life, with the people and in the places that they want to participate in these occupations” [25]. For autistic children, OTs may support their participation and engagement in everyday life activities through a variety of approaches, which include, but are not limited to, addressing sensory processing, self-care, social-behavioural performance and participation in play [26]. For the aforementioned therapies, service delivery may vary based on the provider and goals of the therapy.

Guided by the TFA constructs, the objective of this mixed methods study was to explore how parents of autistic children in Canada perceive the acceptability of pediatric autism services (OT, SLP, ABA). This work was part of a larger study that also explored autistic peoples’ and clinicians’ perspectives on the same services [27].

## Methods

### Positionality Statement

This survey was conducted as part of the first author’s (IC) master’s thesis. IC is a non-autistic researcher focusing on autism health services delivery. CW was one of the Autistic advisors in the Project Advisory Group. She has an M.A. in Critical Disability Studies and has consistently been carrying out a variety of professional roles in autism and accessibility since graduating with her M.A. in 2017. The other authors are all non-autistic scientists who also have clinical training (medicine, psychology, occupational therapy). Together, we acknowledge that discussions about therapies accessed by autistic children are nuanced and can be emotionally laden, and we aim to use this work to respectfully engage with that nuance. Our aim is to identify key values that will inform dialogue about the acceptability of therapies and promote wellbeing among autistic children and their families.

### Advisory Group

A Project Advisory Group (PAG) advised on the development, dissemination and analysis of the survey. This committee was composed of four autistic people, three parents of autistic children and youth (recruited from larger advisory groups at the investigators’ institutions) and three clinicians (recruited from the investigators’ networks), all within the province of Ontario. All members received an honorarium for their participation in the PAG. Due time constraints associated with completing a master’s thesis, fully participatory research, where researchers and community members shared equal decision-making, was not feasible. In such cases, advisory groups are an important alternative [28]; however, their use requires clear communication about their role and expectations. The expectations and timelines of the advisory group were communicated to members of the PAG, in addition to potential limitations of the project (e.g., deadlines for the master’s thesis) [2]. Four meetings were conducted with the PAG: two focused on survey development and two on contextualizing results. At the first meeting, PAG members were familiarized with each of the seven constructs of the TFA and led through a discussion about what they believed was important to capture through survey questions for each of the constructs. Following the first meeting, a draft survey was created and distributed to PAG members for their asynchronous review. The second survey meeting focused on a discussion related to these draft questions, and how they could be improved to better capture lived experiences. The survey questions were updated following discussion, and the final draft of the survey, including the informed consent form, was emailed to PAG members to elicit their feedback on survey content, accessibility, ease of understanding, and language. At the two meetings focused on contextualizing the results, a high-level overview of both qualitative and quantitative findings was shared with members to ascertain the extent to which the results aligned with or diverged from their lived experiences. Discussions at the PAG prompted iterative analysis, and returning to qualitative analysis informed by members’ experiences and perspectives. Throughout the project, a text-based meeting platform, one-on-one meetings, and asynchronous participation (i.e., emails rather than meetings) were offered to facilitate participation.

### Survey Design

As no validated questionnaires relating to the acceptability of autism services have been published, a novel survey was constructed with input from the PAG. The seven constructs of the TFA were used to structure the survey. The survey was created using REDCap, a secure electronic data capture tool [29], and was pilot tested with an autistic person, a parent, and a clinician for language use and clarity. Ethics approval was obtained through a tertiary pediatric hospital and university, and all methods were carried out following institutional guidelines.

Demographic information (age, gender, race/ethnicity, income, and province/territory of residence) was captured. For each therapy type, a broad definition was provided, and respondents answered a set of questions on a Likert-type scale and free-text questions (survey and related definitions available in Appendix A). For each of the seven constructs of the TFA there were 2–3 questions, with Likert-style response options of Strongly Agree, Agree, Neutral/No Opinion, Disagree, and Strongly Disagree. At the end of each section related to one of the therapy types, respondents were asked two free-text questions: 1) “What do you think the purpose of [therapy type] is for autistic children? and 2) “In your opinion, is [therapy type] an acceptable therapy for autistic children? Why or why not?”. Free-text questions were suggested by the PAG to allow respondents further explain their perspectives if desired. The survey collected information on four therapeutic approaches: SLP, OT, ABA and physiotherapy. Respondents reported low engagement and familiarity with physiotherapy; therefore, this approach is excluded from reporting.

### Participants

Parents of autistic children were recruited to complete the survey using social media, snowball sampling, listservs, and newsletters of national and provincial autism organizations. Respondents were asked if their child(ren) received a diagnosis of autism spectrum disorder. Other inclusion criteria were that respondents needed to live in Canada, have access to the Internet, and be able to read and write in English, or access supports (i.e., another individual) that could facilitate survey completion. Importantly, the TFA allows for the evaluation of perceived acceptability from those who have not engaged in the intervention, and we sought parental perspectives irrespective of whether their child had participated in a specific therapy. Informed consent was obtained and documented through the online survey, as respondents had to read and agree to the consent page to participate and navigate to the main survey page. From pilot testing, the survey was estimated to take 20–30 minutes to complete. At the end of the survey, respondents had the option entering their email address to be entered into a raffle for one of 20 $50 gift cards, and their email addresses were not linked to their responses. Recruitment took place between January 27^th^ 2022 and August 25^th^ 2022. No measures were included to prevent potential fraudulent responses to the survey [30].

### Data Analysis and Integration

A participant’s responses were included in the final analysis if they completed the Likert questions for at least one of the included therapies (16 questions). Descriptive statistics were calculated for the demographic data. For each therapy type, a composite sub-score was created based on responses to the scale questions; the highest possible sub-score (most acceptable) was 64 and the lowest sub-score (least acceptable) was zero. Creating a composite score from Likert data is supported when measuring more abstract constructs like acceptability [31]. A measure of internal consistency, Cronbach’s alpha, was calculated for each therapy sub-score, which is recommended when producing a composite score from survey data [31]. The alpha coefficient is between zero and one, and describes the interrelatedness of items of a survey [32]. Coefficients closer to one are indicative of higher internal consistency. The Cronbach’s alpha for OT was 0.87, for SLP was 0.85, and for ABA 0.88, all representing a satisfactory measure of internal consistency.

Guided by the seven TFA constructs, free text responses were analyzed following deductive content analysis [33]. NVivo was used to collate and organize responses. Deductive content analysis is useful when seeking to extend or validate an existing framework like the TFA. Content analysis includes three phases: preparation, organizing and reporting [34]. In the preparation phase, the free-text responses were read and annotated by the researcher (IC). In the organizing phase, responses were categorized based on the the TFA, with subcategories created based on the included quantitative survey questions. IC and MP met biweekly to discuss coding and the analysis. As previously described, qualitative analyses were reported to the PAG for discussion and member checking, which prompted further engagement and analysis of the data in order to enhance credibility [35].

Following a mixed methods approach, the qualitative and quantitative strands were collected concurrently, and their results integrated through triangulation [36,37]. Narrative synthesis (weaving together qualitative and quantitative findings) [38] and joint displays, which are visual representations of qualitative and quantitative data integration, were used to integrate qualitative and quantitative strands [38]. Illustrative quotes from qualitative analysis are included within the joint displays.

## Results

### Participant Characteristics

The survey was opened by 101 potential respondents; of those, 87 parents completed the demographic information and at least one set of the scale questions and were included in the analysis (attrition rate: 13.9%). As multiple sampling methods were used, the response rate cannot be calculated. There was no systemic screening completed for evidence of fraudulent responses; however, there were no incoherent responses nor responses submitted in close succession. Nearly 20% of parent respondents (*n* = 16) identified that they had more than one autistic child, and 12 parents self-identified as autistic (13.8%) (Table 1). Most parent respondents had children who engaged with OT (85.1%) or SLP (82.8%). Fewer had engaged in ABA (47.1%). The ages of parent respondents are visualized using a histogram (S1 Fig).

**Table 1:** Descriptive Statistics of Parent Respondents (*n* = 87).

	n	%	Median	Range
Age (years)	87		44	28-70
Income Above $100,000	54	62.1		
				
**Gender**				
Woman	77	88.5		
Man	4	4.6		
Non-Binary	3	3.4		
Other, self-described	2	2.3		
Prefer not to Answer	1	1.1		
**Location**				
Ontario	16	18.4		
Saskatchewan	19	21.8		
Manitoba	8	9.2		
Alberta	2	2.3		
Prince Edward Island	2	2.3		
British Columbia	1	1.1		
**Highest Level of Education**				
High School	6	6.9		
Bachelor’s Degree	30	34.5		
Master’s Degree	26	29.9		
Doctorate/PhD or higher	6	6.9		
College Diploma	4	4.6		
Trade School	10	11.5		
Other	5	5.7		
Also autistic	12	13.8		
Also clinician	13	14.9		
**More than One Autistic Child**				
Two Autistic Children	14	16.1		
Three Autistic Children	2	2.3		
**First Autistic Child**				
Age of Child’s Diagnosis (years)	87		4	1-35
Current Age of Child (years)	72		12	2-28
**Second Autistic Child**				
Age of Diagnosis (years)	14		4.5	1-12
Current Age (years)	14		7	2-22
**Third Autistic Child**				
Age of Diagnosis (years)	2		2.5	2-3
Current Age (years)	2		9	4-14
**Child Occupational Therapy**				
Respondents whose children participated in OT	74	85.1		
Age beginning therapy (years)	83		3	0-20
Duration of Therapy (years)	82		2	0-16
Funding Type				
Private	38			
Government Funding	45			
School-Funded	17			
Don’t Know	2			
**Speech Language Pathology Services**				
Respondents whose children participated	72	82.8		
Age beginning therapy (years)	77		3	0-14
Duration of Therapy (years)	76		3	0-16
Funding Type				
Private	46			
Government Funding	50			
School-Funded	21			
Don’t Know	1			
**Applied Behavior Analysis**				
Respondents whose children participated	41	47.1		
Age beginning therapy (years)	43		4	1-18
Duration of Therapy (years)	42		2	0-12
Type of ABA				
DTT	12			
EIBI	21			
NDBI	4			
Group-Based	13			
Parent Training/Consultation	23			
Not sure	2			
Funding Type				
Private	15			
Government Funding	34			
School-Funded	1			
Don’t Know	1			

*Note.* DTT = discrete trial training; EIBI = early intensive behavioural intervention; NDBI = naturalistic developmental behavioural intervention

### Acceptability Scores

S2 Fig provides a boxplot of the composite perceived acceptability sub-scores for respondents. The median SLP acceptability sub-score was 41.5 (*Inter-quartile range (IQR) = 10.5*, *n* = 78), the median OT acceptability sub-score was 40 (*IQR = 13*, *n* = 87), and the median ABA sub-score was 28 (*IQR = 14*, *n* = 77). Significant between group differences were identified using one-way analysis of variance tests [F (2, 239) = 43.4, p < 0.01]. Among those who identified accessing a therapy, the median acceptability scores were 42 (*IQR = 10.25, n = 64*) for SLP, 40.5 (*IQR = 11.75, n = 74*) for OT, and 30 (*IQR = 18, n = 35*) for ABA. There were no significant group differences between participants who had access to therapy and those who did not for both OT and SLP; however, this difference was significant for ABA (*t =* 3.36, *p* < 0.01). The median acceptability score for parents whose children had not accessed ABA was 25.5 (*IQR* = 12.75, *n* = 42).

### Narrative Synthesis

Qualitative analysis identified codes predominantly in three TFA constructs (*Ethicality*, *Perceived* *Effectiveness*, and *Self*-*Efficacy*), with few to no codes identified under other TFA constructs. Greater analytic attention was paid to these three constructs as they generated richer responses with greater engagement of respondents, thus suggesting their strong resonance. These are described in more detail below along with Likert responses. Likert responses for other TFA domains are available in Appendix A.

### Ethicality

The TFA defines *Ethicality* as “the extent to which an intervention has good fit with an individual’s value system” [18]. For OT and SLP, most parent respondents selected ‘Agree’ or ‘Strongly Agree’ that the goals (OT: 78.2%, SLP: 85.9% ‘Agree’ or ‘Strongly Agree’) and methods (OT: 75.9%, SLP: 83.4% ‘Agree’ or ‘Strongly Agree’) of these services were in line with their value systems (Table 2). For ABA, 31.2% of parents selected ‘Agree’ or ‘Strongly Agree’ that the goals of this service were aligned with their value systems, and 29.9% selected ‘Agree’ or ‘Strongly Agree’ when asked if the methods of ABA aligned with their personal value systems.

**Table 2:** Joint Display of Parent Perspectives on Ethicality, n (%).

		*From my understanding, the [goals/methods] of [therapy type] align with my personal value systems*					Ethicality Categories & Illustrative Quotes
		*Strongly Disagree*	*Disagree*	*Neutral*	*Agree*	*Strongly Agree*	
**OT** **(*n* = 87)**	Goals	1 (1.1)	2 (2.3)	16 (18.4)	43 (49.5)	25 (28.7)	Affirming of Neurodivergence: *“To teach them the skills needed to participate in the world in a way that allows them to be who they are (i.e., it doesn’t try to change them).”*
	Methods	1 (1.1)	3 (3.4)	17 (19.5)	40 (46)	26 (29.9)	
**SLP (*n* = 78)**	Goals	1 (1.3)	2 (2.6)	8 (10.3)	41 (52.6)	26 (33.3)	Potential Harms “*A great deal of emphasis and urgency is placed in verbal speech, and many [SLPs] fail to understand the necessity and functionality of autistic echolalia and therefore dismiss it, which does great harm to the child.”*
	Methods	1 (1.3)	4 (5.1)	8 (10.3)	40 (51.3)	25 (32.1)	
**ABA** **(*n* = 77)**	Goals	30 (39)	8 (10.4)	15 (19.5)	12 (15.6)	12 (15.6)	Potential Harms *It teaches kids that it is not ok to be who they are and that they have to hide who they are. Can you imagine how that feels? It teaches compliance without supporting or accommodating their needs, which is what needs to be done.*
	Methods	31 (40.3)	7 (9.1)	16 (20.8)	11 (14.3)	12 (15.6)	

*The quotes represent exemplary responses, by qualitative category and type of therapy, and are not representative of all quotes for a type of therapy.*

Analysis of free-text responses identified three main topics under the TFA construct of *Ethicality*: 1) *concerns about potential harms or negative experiences* (particularly related to behavioural goals and principles), 2) *the importance of neurodiversity-affirming services,* and 3) *mixed views on behavioural principles*.

The first topic, potential harms or negative experiences, included possible negative occurrences and outcomes from therapies. Across all three therapy types, parents provided examples of negative experiences they had witnessed (e.g., anxiety, trauma, distress) or wrote about harms they had learned about from autistic adults. One parent shared that for OT to be acceptable, it must draw on the child’s strengths and promote autonomy:

*Yes if it is strength based and not looking to override a sense of self or autonomy/consent for the goal of compliance as a benchmark of success.*

Nested within potential harms, some respondents specifically critiqued what they perceived to be the harms that could be caused by ‘behavioural goals or principles’. Some parent respondents were critical of goals that promoted neurotypical behaviours, such as making the child appear ‘normal’ or encouraging them to fit into neurotypical society. For example, one parent wrote that the purpose of ABA from their view was:

*To train autistic children in socially acceptable behaviour so other people aren’t made uncomfortable by [them] and they fit in better.*

This parent’s response suggests an ethical tension related to changing a child’s behaviour to make neurotypical people more comfortable. Other parent respondents described potential harms of aiming to change behavior through therapies, which included disempowerment of the child, removal of self-soothing or coping mechanisms, trauma, and loss of happiness or sense of self. Some parents identified concerns specific to use of behavioural approaches, such as rewarding a child for displaying a specific behaviour (external reinforcement). When asked about the purpose of ABA, one non-autistic parent wrote:

*I believe the purpose of ABA is to train [a] child using extreme positive reinforcement. It attempts to change a child. Adults who underwent ABA often consider it abusive.*

This parent was influenced by the reports of autistic people who experienced ABA therapy. Ideas of ‘training’ and ‘changing’ were perceived as harmful.

The second topic of the importance/value of neurodiversity-affirming services highlighted what affirming practices for neurodivergent children should look like. As one autistic parent wrote about OT:

*Yes, it helps them accomplish goals by teaching new skills. It is not focused on suppressing who they are, coercing them or forcing them to change to make other people more comfortable even if there are huge mental health costs to the child.*

About the acceptability of SLP, one non-autistic parent shared:

*Hopefully this is done more in a neurodiversity affirming model which doesn’t go to remove autistic traits but aid in communication and well-being of [the] individual.*

Other respondents described that incorporation of alternative and augmentative communication devices in SLP aligned with neurodiversity-affirming approaches by honouring different communication methods.

Many respondents viewed neurodiversity-affirming approaches to therapy as aligning with their personal ethics. Respondents valued accepting the child for who they are, providing accommodations, and not attempting to reduce observable autistic traits.

The last topic under the construct of Ethicality reflected *mixed views* on *behavioural principles*, particularly around the goal of ‘behaviour change’ and the use of behavioural approaches in therapies. Respondents had differing opinions on whether these goals and approaches aligned with their personal values and beliefs. Some viewed behavioural approaches, often discussed in relation to ABA, as effective for teaching new skills and reducing ‘negative’ behaviours, such as task refusal, self-harm/injury, and elopement, whereas others questioned their appropriateness or ethicality. One non-autistic parent described that the purpose of ABA was:

*To help mitigate negative behaviours and building new skills, to gain the skills to lead an independent life.*

Respondents also identified a broad set of skills, including toileting, putting on shoes, communication, joint attention, play, social skills, motor development, and sensory integration that could be learned through ABA. Conversely, some parents were critical of behavioural goals that promoted neurotypical behaviours, such as making the child appear ‘normal’ or encouraging them to fit into neurotypical society, as described above under *potential harms and negative experiences*.

Overall, parent respondents identified that *Ethicality* as a key aspect of acceptability of autism services. Respondents perceived that therapies could cause harms, and many discussed the importance of neurodiversity-affirming approaches. Respondents presented mixed views on the use of behavioural principles to reach goals.

### Perceived Effectiveness

This construct is defined as “the extent to which the intervention is perceived as likely to achieve its purpose” [18]. S3 Fig presents a visual integration of qualitative and quantitative responses. For OT, SLP, and ABA, most parent respondents selected ‘Agree’ or ‘Strongly Agree’ for the statement “I think that [therapy type] can teach new skills to autistic children”. The other statement was “The skills that are emphasized in [therapy type] are not meaningful or impactful in the daily lives of autistic children”. Over 75% of parent respondents selected ‘Disagree’ or ‘Strongly Disagree’ for this statement for OT and SLP services, while comparatively fewer (30%) selected ‘Disagree’ or ‘Strongly Disagree’ for ABA.

Two topics within the construct of *Perceived* *Effectiveness* were identified in the parent free text responses: *importance of seeing visible impact/progress* and *therapist fit.* Some parents described seeing notable successes of therapies through goal achievement (e.g., learning practical skills) or improved child well-being (e.g., growing independence, reduced anxiety). For example, one non-autistic parent wrote about the perceived effectiveness of therapies for their child:

*As controversial as ABA therapy is, its rapid effectiveness with parent involvement is apparent when compared to reaching similar goals in OT and ST [speech therapy] settings. We did OT and ST 2x/week before ABA with very slow or no results. Of course, this wouldn’t be the same for every child.*

This quote suggested that the respondent placed value on the relative speed of goal attainment. Other parents did not notice progress or changes for their child. For example, one parent wrote:

*Therapies are expensive and I often hear parents get discouraged and say therapies are ineffective.*

Thus, some parent respondents described being discouraged from continuing a therapy or stopping it altogether because the therapy was expensive and lacked a perceptible impact.

The second category was the importance of therapist fit. Across all therapy types, some parents expressed that the acceptability of a service was dependent on the therapist delivering it. There were few responses that spoke to what constituted a strong ‘fit’ with families, which expressed the role of positive, respectful relationships. Responses in this category highlighted that parents’ views of acceptability were contingent on how they viewed the therapist and their approach, exemplified in the quote below about SLP services:

*If they (SLP and client) do not mesh well nothing gets accomplished.*

Another parent who had also engaged with SLP services for their child provided a more detailed experience with their therapist:

*It is also tricky to know for all…therapies if my personal experiences were related to the therapy itself or if my experience was more related to the therapist. For example, I had SLPs that worked with my son that didn’t seem to pay any attention to our family’s goals. They were naive at best and mean at worst.*

In summary, the perceived effectiveness, demonstrated both by progress attained through a therapy and the fit with the therapist was identified as an important feature of acceptable therapies by parent respondents.

### Self-Efficacy

This construct is defined by the TFA as “the participant’s confidence that they can perform the behaviour(s) required to participate in the intervention” [18]. Respondents were asked two Likert questions related to this construct. The first question asked respondents to indicate their agreement that the expectations for children’s participation in the therapy are reasonable. For OT and SLP, most respondents selected ‘Agree’ or ‘Strongly Agree’; for this statement (78.1% *n* = 68, and 76.6%, *n* = 60, respectively). For ABA, there was more variation in the level of agreement; almost 30% of respondents selected ‘Agree’ or ‘Strongly Agree’, and almost half of respondents selected ‘Disagree’ or ‘Strongly Disagree’. The second self-efficacy question asked respondents to indicate their level of agreement as to whether parents could complete tasks as directed by a therapist. More than half of respondents selected ‘Agree’ or ‘Strongly Agree’ for OT and SLP. For ABA, nearly half of respondents selected neutral for this statement, and nearly 40% selected ‘Agree’ or ‘Strongly Agree’ (S4 Fig).

The qualitative category under this construct was the *importance of therapists/practitioners engaging with parents*. Responses emphasized the importance of teaching parents or helping them understand how to support their child, particularly in areas such as their communication strategies. As one parent described about the purpose of OT:

*I was able to implement these techniques to help my child make improvements in the global gross motor skills delay.*

This category spanned all therapies. One parent specifically identified their desire for more parent engagement in the SLP services they received. Other parents also described the role of therapists in helping them understand their child’s needs and equipping them with the tools to support their children. As one parent shared about the goal of SLP approaches:

*To help parents get different tools to communicate with their kid.*

Both examples demonstrated parents’ beliefs about the importance of a good fit between the child, family, and therapist for a therapy to effectively and acceptably achieve its intended purpose. In summary, parents’ perspectives on their self-efficacy with respect to autism services was influenced by working with therapists who effectively engaged them, helping them to understand their child and their needs, and enabling their adoption of techniques beyond designated clinical visits.

## Discussion

Perceptions of therapeutic services available to autistic children have been the subject of much academic and popular discussion. With the shifts in how autism is conceptualized, and in turn, what therapies and therapeutic targets are appropriate for autistic children [39], understanding current parent perspectives on these therapies is essential. Here, we present findings from 87 parents who responded to a survey that explored their views on the acceptability of OT, SLP services, and ABA-based therapies. The *Ethicality* of services was a critical influencing factor of acceptability, and respondents shared concerns about potential harms or negative experiences, varying perspectives on the use of behavioural techniques and changing behaviour, and the importance of services affirming neurodivergence. Neurodiversity-affirming therapeutic practice has been described as therapy that does not attempt to reduce or eliminate the diverse neurological processing, ways of being or behaviours that are viewed as autistic (e.g., self-stimulatory behaviour) [40]. Related to the TFA constructs of *Perceived Effectiveness* and *Self-efficacy*, parents’ views on acceptability were influenced by observing visible outcomes for their child (e.g., skill acquisition, increased independence) and by the quality of their relationship/interaction with the therapist.

We identified two linked topics under the construct of *Ethicality*: potential harms/negative consequences of therapies and the importance of affirming neurodivergence. Working to affirm neurodivergence was identified by parent respondents as an important feature of acceptable therapies and may support the long-term mental health of autistic children. Conversely, respondents also described the potential for harm through failing to affirm neurodivergence. This was most often stated about ABA, though less than half of parent respondents had direct experience with ABA. Importantly, there was a significant difference in the acceptability scores of parents whose children had participated in ABA, versus those whose children had not. Parents whose children had accessed an ABA-based therapy had a median acceptability score that was higher than parents who did not access ABA, suggesting that parents who did not have personal experience with ABA might have been informed by external sources.

Further, a key concern was the potential for any therapy to promote adoption of neurotypical norms. A recent survey study of 235 autistic adults identified similar findings as our parent survey, in that respondents viewed goals based on neurotypical normalization to be unacceptable [41]. Respondents from this same survey approved of goals that focus on quality of life, safety, and autistic interactions [41]. In another survey, autistic adults who reported personal and external (friends, family, society) autism acceptance had lower predicted depressive symptoms, suggesting that autism acceptance can be a protective factor against depression [42]. In a more recent survey study about theoretical models and intervention goals, both autistic and non-autistic respondents supported interventions that aim to change society, create supportive environments for autistic people, and promote well-being; however autistic respondents were more likely to support the neurodiversity movement [43]. In a qualitative analysis of an open-ended survey completed by autistic adults, researchers identified support for therapy goals related to autonomy, self-advocacy and interdependence [44]. As identified, therapies should promote personal and external acceptance of autistic identities [42], which contrasts from historical deficit-focused approaches to therapy [45].

Respondents also identified concerns about potential harms resulting from behavioural goals and approaches. Some parent respondents were critical of behavioral techniques, such as the use of external reinforcement; however, there was more variability amongst parent respondents on the acceptability of changing behaviors as a goal of therapy. Parents who endorsed behavior change mentioned skill-building, while others conveyed concerns about the potential harms of promoting adherence to neurotypical standards. This general wariness toward ‘behavioral approaches’ requires more attention, especially considering the differencing perspectives of our sample based on their own personal experiences of ABA. Learning processes are linked to behavioral principles [46] and behavioral concepts are present across all therapy types and informally in other settings, such as the use of positive reinforcement in schools [47]. Accordingly, our work shows an urgent need for more specificity in understanding what parents understand to be a behavioural approach, given the many possible interpretations of behavioural techniques and their broad application.

Our results suggest heightened parental awareness of neurodiversity and the perspectives of autistic people on services accessed by autistic children relative to earlier studies [39]. A 2012 study identified that some parents endorsed neurodiversity sentiments, such as describing children’s behaviour as natural cognitive variations rather than a disorder, sharing positive comments about autism, and rejecting ideas that their child’s diagnosis was negative or a burden, despite pursuing treatment options that may conflict with a neurodiversity paradigm [48]. A more recent study identified broad support for the neurodiversity movement amongst autistic people, researchers, professionals and parents/caregivers [49]. Another study asked parents to identify the ‘best things’ about their autistic child, and the authors concluded that ideas about strength and resilience were aligned with a neurodiversity model [50]. Parents who were interviewed about their autistic child’s echolalia identified it as an aspect of their child’s identity, and one they did not want to address with intervention [51]. These articles, and the findings from this survey, highlight that parents may increasingly be engaging with neurodiversity-informed views of autism.

Parent respondents had important input as to the importance of observing changes through therapies, as well as therapist and parental roles in bringing about these changes. Under the TFA construct of *Perceived Effectiveness*, some parents described the importance of seeing visible impact or changes from a therapy. A subset of parents identified how ABA-based therapies produced more apparent improvements than OT or SLP services. This finding suggests that a parent’s expectations for what a service is to achieve plays a role in its’ acceptability. Other research has identified that parent expectations may play a role in their evaluation of therapies, specifically social-communication therapy [52]. Further, parent respondents feel that acceptability is contingent on the therapist’s ability to work with both their child (*Perceived Effectiveness*), and to empower parents in implementing therapeutic strategies (ex., providing tools, helping parents understand child’s needs) (*Self-Efficacy*). It is critical that therapists communicate clear and realistic expectations of a service, expected impacts, and what role they as parents can play. As identified by respondents, the role of therapists in helping parents understand their child’s needs and behaviours are integral to successful delivery. Parents understandably want to see an impact from the services their child is accessing; however, this occurs in the context of continued evolution of intervention targets to focus more on strengths, pleasure, and well-being and rather than on deficits, in line with neurodiversity-affirming practices [53].

There are many implications from this study. Our results suggest a need to meaningfully engage the voices and experiences of parents/care partners when designing therapies for autistic children, in addition to the lived experiences of autistic individuals. Calls to reframe what constitutes an ‘effective’ therapy for autistic children, measure autistic-prioritized outcomes, and partner with autistic people have been made, in the spirit of aligning autism therapies with the neurodiversity paradigm [53]. Other neurodiversity-affirming clinical practices include incorporating the lived experiences of autistic adults into clinical training, encouraging the child to express their needs and preferences in therapy, and identifying goals that support the child’s quality of life through shared decision-making with them and their family [54]. Future work should incorporate trauma-informed, good-faith collaboration between autistic people, parents, teachers, clinicians and seek to gain a shared understanding of key areas identified in this survey, such as behaviour approaches, behavior change, and neurodivergence-affirming therapy. There is also a need to create and evaluate materials that make children and families aware of different therapies and facilitate a shared, current, and comprehensive understanding of their methods and aims.

This study has several limitations. An online, cross-sectional survey introduces selection bias where those who have strong feelings about the topic may be more likely to participate. Because of the cross-sectional nature of the work, we were unable to probe for further clarification on terms like ‘behavioral approaches’. A variety of approaches were included under the umbrella of ABA (ex. EIBI, DBDT); however, we did not analyze perspectives on ABA based on specific type of therapy accessed, which could have influenced a respondent’s experience. Our sample was lacking in racial/ethnic diversity, and it is crucial that perspectives on acceptability of these therapies are informed by parents who can speak to the cultural safety of services. Autistic perspectives are essential to ensuring that services align with acceptable, neurodiversity-affirming practices; however, despite efforts to recruit autistic survey respondents, we struggled to reach enough autistic respondents to adequately compare their perspectives with that of the parent sample.

In conclusion, across this sample parents of autistic children, we identified *Ethicality* appears to play a meaningful role in shaping acceptability. The perspectives provided by parents that services should not cause harm and should work to affirm autistic identities echoes themes/ideas emphasized within autistic-led advocacy. Additionally, therapists/practitioners have vital roles to play in supporting parents to engage with their child by providing strategies, explaining their approach, and communicating realistic outcomes and goals of services. Moving forward, it is crucial that therapists and those involved in receiving therapies (both children and parents) have a shared understanding of the values, goals and preferences that are integrated into services and to continually evaluate together how a certain approach is meeting those needs and goals.

## Supporting information

S1 Fig Histogram of Respondents’ Ages. (DOCX)

S2 Fig Composite Sub-Scores of Parent Acceptability Responses (OT, Occupational Therapy; SLP, Speech Language Pathology; ABA, Applied Behaviour Analysis). Note. With box plots, the lowest and highest boundaries of the box represent the 25th and 75th percentile, respectively. The line within the box represents the median, and the ‘X’ represents the mean. The highest and lowest whiskers represent the 90th and 10th percentiles, and points outside of these whiskers are outliers.
(DOCX)

S3 Fig Joint Display of Parent Perspectives on Perceived Effectiveness, n (%). (DOCX)

S4 Fig Joint Display of Parent Perspectives on Self-Efficacy, n (%). (DOCX)

S1 File Parent Responses. (DOCX)
