# Supplementary material for: Parent perspectives on services available to autistic children in Canada: A mixed methods survey study
Source: PLoS One. 2026 Aug 20;21(8):e0353678. doi: 10.1371/journal.pone.0353678 (PMC13492781; doi:10.1371/journal.pone.0353678)
Supplement: Supplementary file 2 [file pone.0353678.s002.docx]

Figure S2. Composite Sub-Scores of Parent Acceptability Responses (OT, Occupational Therapy; SLP, Speech Language Pathology; ABA, Applied Behaviour Analysis)

*Note. With box plots, the lowest and highest boundaries of the box represent the 25^th^ and 75^th^ percentile, respectively. The line within the box represents the median, and the ‘X’ represents the mean. The highest and lowest whiskers represent the 90^th^ and 10^th^ percentiles, and points outside of these whiskers are outliers.*
