# Supplementary material for: Parent perspectives on services available to autistic children in Canada: A mixed methods survey study
Source: PLoS One. 2026 Aug 20;21(8):e0353678. doi: 10.1371/journal.pone.0353678 (PMC13492781; doi:10.1371/journal.pone.0353678)
Supplement: Supplementary file 3 [file pone.0353678.s003.docx]

Figure S3. Joint Display of Parent Perspectives on the Perceived Effectiveness, n (%)

|  | ***I think that [therapy type] can teach new skills to autistic children*** | | | | | **Perceived Effectiveness  Qualitative Categories** |
| --- | --- | --- | --- | --- | --- | --- |
|  | *Strongly Disagree* | *Disagree* | *Neutral* | *Agree* | *Strongly Agree* |  |
| **OT**  **(*n*=87)** | 1  (1.1) | 3  (3.4) | 10  (11.5) | 32  (36.8) | 41  (47.1) |  |
| **SLP**  **(*n*=78)** | 1  (1.3) | - | 10  (12.8) | 31  (39.7) | 29  (37.2) |  |
| **ABA**  **(*n*=77)** | 10  (13) | 2  (2.6) | 17  (22.1) | 28  (36.4) | 20  (26) |  |
|  | ***The skills that are emphasized in [therapy type] are not meaningful or impactful in the daily lives of autistic children (Reverse Scored)*** | | | | |  |
|  | *Strongly Agree* | *Agree* | *Neutral* | *Disagree* | *Strongly Disagree* |  |
| **OT**  **(*n*=87)** | 4  (4.6) | 5  (5.7) | 7  (8) | 30  (34.5) | 41  (47.1) |  |
| **SLP**  **(*n*=78)** | 2  (2.6) | 6  (7.7) | 9  (11.5) | 26  (33) | 35  (44.9) |  |
| **ABA**  **(*n*=77)** | 19  (24.7) | 7  (9.1) | 28  (36.4) | 10  (13) | 13  (16.9) |  |

Therapist Fit

It helps meet a specific need for autistic children, assuming the therapist is trained in working with them & respectful to the child and family

Seeing Visible Impact or Progress

Based on my child's progression when ABA was introduced, the results were rapid compared to ST and OT. I personally found this the most effective and diverse therapy for my child
