# Supplementary material for: Parent perspectives on services available to autistic children in Canada: A mixed methods survey study
Source: PLoS One. 2026 Aug 20;21(8):e0353678. doi: 10.1371/journal.pone.0353678 (PMC13492781; doi:10.1371/journal.pone.0353678)
Supplement: Supplementary file 4 [file pone.0353678.s004.docx]

Fig S4.

Figure S4. Joint Display of Perspectives on the Self-Efficacy, n (%)

|  | ***The expectations for autistic children’s’ participation in [therapy type] are reasonable*** | | | | | **Self-Efficacy Qualitative Categories** |
| --- | --- | --- | --- | --- | --- | --- |
|  | *Strongly Disagree* | *Disagree* | *Neutral* | *Agree* | *Strongly Agree* |  |
| **OT**  **(n = 87)** | 2  (2.3) | 9  (10.3) | 8  (8.2) | 53 (60.9) | 15  (17.2) |  |
| **SLP**  **(n = 78)** | 1  (1.3) | 3  (3.8) | 14  (17.9) | 46  (59) | 14  (17.9) |  |
| **ABA**  **(n =77)** | 25  (32.5) | 12  (15.6) | 18  (23.4) | 14 (18.2) | 8  (10.4) |  |
|  | ***If asked by a [therapist type], I believe parents could adequately support their child’s participation in [therapy]*** | | | | |  |
|  | *Strongly Disagree* | *Disagree* | *Neutral* | *Agree* | *Strongly Agree* |  |
| **OT**  **(n = 87)** | 1  (1.1) | 8  (9.2) | 20  (23) | 46 (52.9) | 12  (13.8) |  |
| **SLP**  **(n = 78)** | - | 5  (6.4) | 13  (16.7) | 43  (55.1) | 17  (21.8) |  |
| **ABA**  **(n =77)** | 8  (10.4) | 5  (6.5) | 35  (45.5) | 22 (28.6) | 7  (9.1) |  |

Importance of Therapists Engaging with Parents

[About the purpose of SLP]: To help parents get different tools to communicate with their kid.
