# Supplementary material for: Parent perspectives on services available to autistic children in Canada: A mixed methods survey study
Source: PLoS One. 2026 Aug 20;21(8):e0353678. doi: 10.1371/journal.pone.0353678 (PMC13492781; doi:10.1371/journal.pone.0353678)
Supplement: Supplementary file 5 [file pone.0353678.s005.docx]

File S1 – Parent Responses

Table 1. **Parent** Responses to Likert Survey Items about Occupational Therapy (n (%)) (n = 87)

|  | Strongly Disagree | Disagree | Neutral/No Opinion | Agree | Strongly Agree |
| --- | --- | --- | --- | --- | --- |
| I think that OT can teach new skills to autistic children | 1  (1.1) | 3  (3.4) | 10  (11.5) | 32  (36.8) | 41  (47.1) |
| Considering the costs, the benefits of OT outweigh the negatives | 5  (5.7) | 2  (2.3) | 15  (17.2) | 32  (36.8) | 33  (37.9) |
| Parents of autistic children must give up things in their life for their child to participate in OT (R) | 5  (5.7) | 15  (17.2) | 13  (14.9) | 28  (32.2) | 26  (29.9) |
| I have a good understanding of how OT works | 1  (1.1) | 4  (4.6) | 8  (9.2) | 46  (52.9) | 28  (32.2) |
| It takes too much effort for parents to support their autistic child going to OT (R) | 18  (20.7) | 30  (34.5) | 20  (23) | 11  (12.6) | 8  (9.2) |
| The expectations for autistic children's participation in OT are reasonable | 2  (2.3) | 9  (10.3) | 8  (8.2) | 53  (60.9) | 15  (17.2) |
| If I had a friend with an autistic child, I would not recommend that they look into OT (R) | 44  (50.6) | 18  (20.7) | 9  (10.3) | 9  (9) | 9  (10.3) |
| From my understanding, the methods used in OT align with my personal value systems | 1  (1.1) | 3  (3.4) | 17  (19.5) | 40  (46) | 26  (29.9) |
| OT costs too much money for autistic children and their families (R) | 6  (6.9) | 11  (12.6) | 18  (20.7) | 33  (37.1) | 19  (21.8) |
| I have a good understanding of the goals of OT | 2  (2.3) | 4  (4.6) | 5  (5.7) | 45  (51.7) | 31  (35.6) |
| Autistic children must give up other things in their life to participate in OT (R) | 8  (9.2) | 26  (29.9) | 23  (26.4) | 26  (29.9) | 4  (4.6) |
| From my understanding, the goals of OT align with my personal value systems. | 1  (1.1) | 2  (2.3) | 16  (18.4) | 43  (49.5) | 25  (28.7) |
| The skills that are emphasized in OT are not meaningful or impactful in the daily lives of autistic children (R) | 41  (47.1) | 30  (34.5) | 7  (8) | 5  (5.7) | 4  (4.6) |
| OT takes too much effort for autistic children to participate in (R) | 16  (18.4) | 45  (51.7) | 19  (21.8) | 5  (5.7) | 2  (2.3) |
| If asked by an occupational therapist, I believe parents could adequately support their child's participation in OT | 1  (1.1) | 8  (9.2) | 20  (23) | 46  (52.9) | 12  (13.8) |
|  | Extrem. Positive | Positive | Neutral/ No opinion | Negative | Extrem. Negative |
| I would describe my attitude towards the use of OT with autistic children as … | 33  (37.9) | 39  (44.8) | 10  (11.5) | 3  (3.4) | 2  (2.3) |

Note. (R) indicates that a question has been reverse scored. Questions are truncated to fit into table.

Table 2. **Parent** Responses to Speech-Language Pathology Likert Questions (n = 78) (n (%))

|  | Strongly Disagree | Disagree | Neutral/No Opinion | Agree | Strongly Agree |
| --- | --- | --- | --- | --- | --- |
| I think that SLP services can teach new skills to autistic children | 1  (1.3) |  | 10  (12.8) | 38  (48.7) | 29  (37.2) |
| Considering the costs, the benefits of SLP services outweigh the negatives | 2  (2.6) | 6  (7.7) | 10  (12.8) | 31  (39.7) | 29  (37.2) |
| Parents of autistic children must give up things in their life for their child to participate in SLP services (R) | 5  (6.4) | 13  (16.7) | 13  (16.7) | 34  (43.6) | 13  (16.7) |
| I have a good understanding of how SLP services work |  | 1  (1.3) | 3  (3.8) | 41  (52.6) | 33  (42.3) |
| It takes too much effort for parents to support their child going to SLP services (R) | 14  (17.9) | 39  (50) | 17  (21.8) | 6  (7.7) | 2  (2.6) |
| The expectations for autistic children's participation in SLP services are reasonable | 1  (1.3) | 3  (3.8) | 14  (17.9) | 46  (59) | 14  (17.9) |
| If I had a friend with an autistic child, I would not recommend that they look into SLP services (R) | 28  (35.9) | 16  (20.5) | 9  (11.5) | 10  (12.8) | 15  (19.2) |
| From my understanding, the methods used in SLP services align with my personal value systems | 1  (1.3) | 4  (5.1) | 8  (10.3) | 40  (51.3) | 25  (32.1) |
| Speech-language pathology services cost too much money for autistic children and their families to participate in (R) | 4  (5.1) | 12  (15.4) | 21  (26.9) | 27  (34.6) | 14  (17.9) |
| I have a good understanding of the goals of speech-language pathology services |  |  | 5  (6.4) | 45  (57.7) | 28  (35.9) |
| Autistic children must give up other things in their life to participate in SLP services (R) | 6  (7.7) | 20  (25.6) | 20  (25.6) | 25  (32.1) | 7  (9) |
| From my understanding, the goals of SLP services align with my personal value systems | 1  (1.3) | 2  (2.6) | 8  (10.3) | 41  (52.6) | 26  (33.3) |
| The skills that are emphasized in SLP services are not meaningful or impactful in the daily lives of autistic children. (R) | 35  (44.9) | 26  (33.3) | 9  (11.5) | 6  (7.7) | 2  (2.6) |
| SLP services take too much effort for autistic children to participate in (R) | 17  (21.8) | 35  (44.9) | 15  (19.2) | 9  (11.5) | 2  (2.6) |
| If asked by a speech-language pathologist, I believe parents could adequately support their child's participation in SLP |  | 5  (6.4) | 13  (16.7) | 43  (55.1) | 17  (21.8) |
|  | Extrem. Positive | Positive | Neutral/No Opinion | Negative | Extrem. Negative |
| I would describe my attitude towards the use of SLP services with autistic children as: | 15  (42.9) | 14  (40) | 4  (11.4) | 1  (2.9) | 1  (2.9) |

Note. (R) indicates that a question has been reverse scored. Questions are truncated to fit into table.

Table 3. **Parent** Responses to Likert Survey Items about Applied Behavior Analysis-based Therapy. (n (%)) (n = 77)

|  | Strongly Disagree | Disagree | Neutral/No Opinion | Agree | Strongly Agree |
| --- | --- | --- | --- | --- | --- |
| I think that ABA can teach new skills to autistic children | 10  (13) | 2  (2.6) | 17  (22.1) | 28  (36.4) | 20  (26) |
| Considering the costs, the benefits of ABA outweigh the negatives | 21  (27.3) | 14  (18.2) | 21  (27.3) | 8  (10.4) | 13  (16.9) |
| Parents of autistic children must give up things in their life for their child to participate in ABA (R) | 1  (1.3) | 2  (2.6) | 21  (27.3) | 25  (32.5) | 28  (36.4) |
| I have a good understanding of how ABA works | 2  (2.6) | 3  (3.9) | 8  (10.4) | 29  (37.7) | 35  (45.5) |
| It takes too much effort for parents to support their child going to ABA (R) | 6  (7.8) | 12  (15.6) | 27  (35.1) | 19  (24.7) | 13  (16.9) |
| The expectations for autistic children's participation in ABA are reasonable | 25  (32.5) | 12  (15.6) | 18  (23.4) | 14  (18.2) | 8  (10.4) |
| If I had a friend with an autistic child, I would not recommend that they look into ABA (R) | 16  (20.8) | 9  (11.7) | 20  (26) | 9  (11.7) | 23  (29.9) |
| From my understanding, the methods used in ABA align with my personal value systems | 31  (40.3) | 7  (9.1) | 16  (20.8) | 11  (14.3) | 12  (15.6) |
| ABA costs too much money for autistic children and their families (R) | 3  (3.9) | 7  (9.1) | 24  (31.2) | 12  (15.6) | 31  (40.3) |
| I have a good understanding of the goals of ABA | 1  (1.3) | 3  (3.9) | 9  (11.7) | 25  (32.5) | 39  (50.6) |
| Autistic children must give up other things in their life to participate in ABA (R) | 4  (5.2) | 5  (6.5) | 18  (23.4) | 22  (28.6) | 28  (36.4) |
| From my understanding, the goals of ABA align with my personal value systems | 30  (39) | 8  (10.4) | 15  (19.5) | 12  (15.6) | 12  (15.6) |
| The skills that are emphasized in ABA are not meaningful or impactful in the daily lives of autistic children (R) | 13  (16.9) | 10  (13) | 28  (36.4) | 7  (9.1) | 19  (24.7) |
| ABA takes too much effort for autistic children to participate in (R) | 7  (9.1) | 13  (16.9) | 19  (24.7) | 13  (16.9) | 25  (32.5) |
| If asked by a behavioral analyst, I believe parents could adequately support their child's participation in ABA. | 8  (10.4) | 5  (6.5) | 35  (45.5) | 22  (28.6) | 7  (9.1) |
|  | Extrem. Positive | Positive | Neutral | Negative | Extrem. Negative |
| I would describe my attitude towards the use of ABA with autistic children as | 11  (14.5) | 12  (15.8) | 18  (23.7) | 21  (18.4) | 21  (27.6) |

Note. (R) indicates that a question has been reverse scored. Questions are truncated to fit into table.

Table 4. **Parent** Responses to Likert Survey Items about Physiotherapy. (n = 75) (n (%))

|  | Strongly Disagree | Disagree | Neutral/No Opinion | Agree | Strongly Agree |
| --- | --- | --- | --- | --- | --- |
| I think that PT can teach new skills to autistic children | - | - | 38  (50.7) | 26  (34.7) | 11  (14.7) |
| Considering the costs, the benefits of PT outweigh the negatives |  | 2  (2.3) | 36  (48) | 24  (32) | 13  (17.3) |
| Parents of autistic children must give up things in their life for their child to participate in PT (R) | 2  (2.7) | 9  (12) | 38  (50.1) | 23  (30.7) | 3  (4) |
| I have a good understanding of how PT works | 1  (1.3) | 10  (13.3) | 24  (32) | 26  (34.7) | 14  (18.7) |
| It takes too much effort parents to support their autistic child going to PT (R) | 5  (6.7) | 25  (33.3) | 41  (54.7) | 3  (4) | 1  (1.3) |
| The expectations for autistic children's participation in PT are reasonable | - | 1  (1.1) | 42  (56) | 24  (32) | 8  (10.7) |
| If I had a friend with an autistic child, I would not recommend that they look into PT (R) | 9  (12) | 14  (18.7) | 38  (50.7) | 10  (13.3) | 4  (5.3) |
| From my understanding, the methods used in PT align with my personal value systems | - | - | 36  (58) | 25  (33.3) | 14  (18.7) |
| PT costs too much money for autistic children and their families to participate in (R) | 4  (5.3) | 6  (8) | 45  (60) | 13  (17.3) | 7  (9.3) |
| I have a good understanding of the goals of PT | - | 5  (6.7) | 32  (42.7) | 22  (29.3) | 16  (21.3) |
| Autistic children must give up other things in their life to participate in PT (R) | 2  (2.7) | 13  (17.3) | 44  (58.7) | 13  (17.3) | 3  (4) |
| From my understanding, the goals of PT align with my personal value systems | - | - | 37  (49.3) | 22  (29.3) | 16  (21.3) |
| The skills that are emphasized in PT are not meaningful or impactful in the daily lives of autistic children (R) | 13  (17.3) | 17  (22.7) | 40  (53.3) | 5  (6.7) |  |
| PT takes too much effort for autistic children to participate in (R) | 8  (9.2) | 21  (24.1) | 41  (54.7) | 5  (6.7) | - |
| If asked by a physical therapist, I believe parents could adequately support their child's participation in PT | - | 2  (2.7) | 36  (48) | 32  (42.7) | 5  (6.7) |
|  | Extrem.  Positive | Positive | Neutral | Negative | Extrem. Negative |
| I would describe my attitude towards the use of physical therapy with autistic children as | 12  (16) | 22  (29.3) | 41  (54.7) | - | - |

Note. (R) indicates that a question has been reverse scored. Questions are truncated to fit into table.
