# Supplementary material for: Parent perspectives on services available to autistic children in Canada: A mixed methods survey study
Source: PLoS One. 2026 Aug 20;21(8):e0353678. doi: 10.1371/journal.pone.0353678 (PMC13492781; doi:10.1371/journal.pone.0353678)
Supplement: Supplementary file 6 [file pone.0353678.s007.docx]

**February 8^th^ 2026**

**PLOSOne - Response to Reviewer Comments**

Title: Parent Perspectives on Services Available to Autistic Children in Canada: A Mixed Methods Survey Study

Dear Reviewers,

We thank the reviewers for insightful comments on this article. Below is an itemized list of all comments from each reviewer and our responses. We look forward in hearing back from you.

| Comment | Response |
| --- | --- |
| REVIEWER 1 |  |
| The framing of the first part of the introduction could be updated to highlight that the recent research on autistic adults’ perspectives on services is in direct response to autistic people’s voices being ignored for decades. At least in ABA research, when acceptability/satisfaction is assessed, it is usually from the parent’s (or a clinician’s) point of view; rarely is the perspective of the autistic person considered. While it is of course still important to get parents’ perspectives – especially the kind of in-depth perspectives obtained in this study! – there could be more explanation as to why researchers have been focusing more on autistic voices. | Thank you for this point, the introduction has been updated with critical calls to autistic communities and frameworks that support researchers in addressing gaps in autistic engagement. We have added the following text on page 4: *The voices of autistic adults were not traditionally sought out through research; however a growing number of calls to action (2, 3) and frameworks (4, 5) provide researchers with the tools to engage those with lived experience.* |
| -Another larger interview study with autistic adolescents also found several critiques of “social intervention” practices: Bottema-Beutel K, Mullins TS, Harvey MN, Gustafson JR, Carter EW. Avoiding the “brick wall of awkward”: Perspectives of youth with autism spectrum disorder on social-focused intervention practices. Autism. 2016;20(2):196-206. doi:10.1177/1362361315574888 | We have introduced this important point into the discussion. |
| Another recent survey study looked at autistic and non-autistic individuals’ perspectives on intervention goals. While they did not specifically report on parents, this study may be of interest: Hersh L, Dwyer P, Kapp SK, et al. Community Member Views on Autism Intervention: Effects of Closeness to Autistic People with Intellectual Disabilities And Nonspeaking Autistic People. Autism in Adulthood. 2024;6(3):253-271. doi:10.1089/aut.2023.0202 | Thank you for including this manuscript, it has been added to the discussion to provide more context to our results. |
| Waddington et al. also recently published a qualitative investigation of support goals, which includes input from parents (Waddington H, Jordan P, Scott E, et al. Community perspectives on support goals for young autistic children: A qualitative investigation. Neurodiversity. 2023;1:27546330231218587. doi:10.1177/27546330231218587) | This is an excellent paper and has been included in the introduction. Thank you. |
| -In ABA, researchers/clinicians are supposed to be assessing “social validity,” which includes appropriateness/acceptability of goals, procedures, and outcomes. While this is certainly done way less than it should be, there are many instances of ABA studies where parents have been asked about their satisfaction with the service provided. However, these assessments are often done in very crude ways, usually without validated instruments. Thus, studies often end up reporting something like, “Parents rated it 6.9/8, so it’s great,” when really we don’t have any way of knowing what “6.9” means, nor do we have nuanced detail about what was good versus bad. You allude to this on page 5, but highlighting this in more detail would strengthen the rationale for your study, because your study actually goes into much more nuance.  -Even if social validity assessment in ABA has been a bit of a mess, many researchers likely see social validity as a theoretical framework that incorporates the significance of goals, appropriateness of procedures, and importance of the effects (Wolf, 1978; for an updated framework, see: Snodgrass MR, Chung MY, Kretzer JM, Biggs EE. Rigorous Assessment of Social Validity: A Scoping Review of a 40-Year Conversation. Remedial and Special Education. 2022;43(2):114-130. doi:10.1177/07419325211017295). Therefore, the authors should reconsider saying that no existing research has explored parental perspectives through the lens of a theoretical framework. | Thank you for providing this detail in regards to social validity, this is an important nuance for us to engage with as we explore this topic in the introduction. We have added both of these citations to add further understanding of the role of social validity. |
| -The introduction would also benefit from more explanation of what ABA, OT, and SLP services look like. | We have added a new paragraph providing a high level overview of these services, on page 7.  *The three therapies were chosen for this survey as an Ontario-based survey of parents found these services to be the most popular (80% would choose ABA for their child, 58% would choose SLP, 58% would choose OT) (19). Broadly speaking, the aim of ABA is to teach different skills by encouraging, or reinforcing, behaviours required for that skill (21). ABA is inclusive of early intensive behavioural intervention (EIBI), discrete trial training (DTT) (22), and newer models that integrate a developmental approach, such as pivotal response training and the Early Start Denver Model (EDSM) (23). According to Speech-Language & Audiology Canada, SLPs support individuals with a variety of issues, including speech delays, language delays, swallowing and feeding disorders, cognitive-communication issues, and literacy skills (24). For autistic children, SLPs support developing interpersonal communication strategies that are uniquely suited to a child, such as augmentative and alternative communication (25). According to the Canadian Association of Occupational Therapists, OT “promotes health, well-being, and quality of life by supporting access to, initiation of, and sustained participation in the things that clients want and need to do in their daily life, with the people and in the places that they want to participate in these occupation” (26). For autistic children, OTs may support their participation and engagement in everyday life activities through a variety of approaches, which include, but are not limited to, addressing sensory processing, self-care, social-behavioural performance and participation in play (27).* |
| -On page 8, consider saying no “published” or “validated” survey relating to acceptability of autism services exists, as multiple other studies have used questionnaires to ask about this (eg the one used by Waddington, the “Autism Intervention Attitudes Scale” used by Hersh et al. and Baiden et al.). | Thank you for pointing this out – we have updated the language to more accurately reflect the existing survey landscape. |
| -Were there any inclusion/exclusion criteria related to age of child? | There were no inclusion/exclusion criteria related to the age of the child. |
| -Were any measures taken to ensure data integrity (i.e., to ensure participants were “real” and not fraudulent?). | The issue with survey data integrity has become an increasing issue in the last few years. The REDCap survey platform incorporates a CAPTCHA feature to prevent against bot respondents.  Thankfully, we did not have any flags for fraudulent responses (i.e., responses in rapid succession, nonsensical answers). However, this was not something we systematically screened for at the time of data collection (2022). |
| -Were parents asked about whether children were currently receiving the service and, if not, how long ago their children were enrolled? (Sorry, I couldn’t find the demographics survey in the supplemental materials.) This could impact acceptability ratings. | Parent respondents were asked the age of their child when they began a therapeutic approach and how long this service lasted for (in years). |
| -Table 1 makes it seem like OT was universally associated with neurodiversity-affirming practices, SLP was associated with potential harms, etc. While I can tell from the narrative that this is not the case, this is somewhat confusing. | We have added a footnote to Table 1 that says - *The quotes presented in Table 1 present example responses, by qualitative category and type of therapy, and are not representative of all quotes for a type of therapy.”* |
| -It should be made clearer that parents were asked about services even if their child had never received such services. It would also be helpful, if possible, to look into whether responses differed significantly between those whose children had versus had not participated in the service (especially for ABA since it was about 50/50). A critique of research with autistic adults is that some never had ABA, so those folks’ perspectives are not relevant. (To be clear, I do not agree with this, but it is a critique I’ve seen.) Some might have this critique of parents in this study who are commenting on ABA but whose children have never engaged in it. If not possible to look into this more, this could just be discussed more the discussion. | We have further clarified in the manuscript that a parent’s child did not have to have engaged in a service in order for them to participate in the survey.  We added a brief overview of the acceptability score among people who accessed that therapy and indicated where there are statistically significant differences. |
| Discussion: |  |
| -The authors may also want to consider citing a recent qualitative study of autistic adults’ perspectives on “intervention” goals, as many of these studies are showing that parents’ and autistic adults’ perspectives converge: Schuck RK, Geng A, Doss Y, et al. A qualitative investigation into autistic adults’ perspectives on intervention goals for autistic children. Neurodiversity. 2024;2:27546330241266718. doi:10.1177/27546330241266718 | This study has been added into the discussion, thank you for pointing out to us. |
| -See another recent study about parents’ views of neurodiversity: Dwyer P, Gurba AN, Kapp SK, et al. Community views of neurodiversity, models of disability and autism intervention: Mixed methods reveal shared goals and key tensions. Autism. Published online September 18, 2024:13623613241273029. doi:10.1177/13623613241273029 | We have added this citation to the discussion to emphasize that parents in may have become more familiar with neurodiversity in recent years. |
| REVIEWER 2 |  |
| MAJOR CONCERNS |  |
| 1. Why focus on these three interventions and not others? Have they been chosen because they are one on one interventions and not group ones? Some very important types of interventions seem to have been left out of this work (e.g. parent training, early sociodevelopmental interventions such as the Denver Model, etc.). Please provide a rationale for focusing on these three interventions only. Please also provide summary descriptions of these three types of intervention. | The decision to choose these three interventions was based on the popularity of services as identified by a survey of Ontario-based parents of autistic children (added to introduction), see page 6: *The three therapies were chosen for this survey as an Ontario-based survey of parents found these services to be the most popular (80% would choose ABA for their child, 58% would choose SLP, 58% would choose OT) (19).*  High level definitions of the therapies have been added to a new paragraph, including sub-types of ABA, on page 7, *ABA is inclusive of early intensive behavioural intervention (EIBI), discrete trial training (DTT) (22), and newer models that integrate a developmental approach, such as pivotal response training and the Early Start Denver Model (EDSM) (23).* |
| 2. One possible bias affecting the results is that parents whose child did not benefit from the intervention could also provide answers regarding that type of intervention. Are the ABA related results credible, since only half of the parents had the occasion to engage with this form of intervention? Are the quantitative results different between the participants familiar with ABA (i.e. with their child having benefited from ABA) and the others? | We have further clarified in the manuscript that a parent’s child did not have to have engaged in a service in order for them to participate in the survey.  We added a brief overview of the acceptability score among people who accessed that therapy and indicated where there are statistically significant differences, see page 15:  “*Among those who identified accessing a therapy, the median acceptability scores were 42 (IQR = 10.25, n = 64) for SLP, 40.5 (IQR = 11.75, n = 74) for OT, and 30 (IQR = 18, n = 35) for ABA. There were no significant differences based on those who had and had not accessed OT and SLP; however, this difference was significant for ABA (t = 3.36, p< 0.01). The median acceptability score for parents whose children had not accessed ABA was 25.5 (IQR = 12.75, n = 42).”* |
| 3. I would recommend providing a scatterplot of participants by age, just to get a full picture of the age demography of the sample. Also, it could have been interesting to make the parents assess the approximate date of intervention for their child(ren), to get a grasp of a possible effect of date/time on their responses (maybe more recent interventions are more acceptable ? Regardless of intervention type?). Please provide some additional data if you have some, or maybe just comment on this point in the limitation section? Maybe you could get this info if you cross sociodemographical data from the parents (their age at moment of survey for example) with the « age beginning therapy » variable available in Table 1. Of course this suggestion might be too complex for the qualitative data, but maybe it could be worthy to try it on quantitative data? | We have added a histogram to the appendix of respondent’s ages. No further analysis of age was conducted. |
| 4. Some of the durations of therapy seem to be really extreme (up to 16 years for SLP and OT). Are these profiles associated with peculiar answers or attitudes toward the interventions? The median value is also very interesting, since all intervention types have a median duration of 2 to 3 years, which is more than what is usually recommended or allowed by public fundings (but I come from a French background). | We did not explore differences in responses based on duration of therapy, but recognize that this may be different based on location |
| 5. Page 27, last paragraph of the conclusion. « In conclusion, across this sample parents of autistic children, Ethicality was a key construct shaping acceptability ». It is an interesting view but nothing in the design or the results allow for prioritizing this dimension over Effectiveness and Self-Efficacy. One methodology that could have solved this is to make participants rank the 7 TFA dimensions from most to least important to them, but I don’t think the authors used this kind of methodology? | We acknowledge that we did not include any specific methodology for determining which constructs would or would not be included in the analysis.  This decision was based on the fact that the majority of responses touched on these responses, whereas the other responses did not, as we highlighted in the manuscript.  We have included on page 15: *Greater analytic attention was paid to these three constructs as they generated richer responses with greater engagement of respondents, thus suggesting their strong resonance.*  Line 556 now reads - In conclusion, across this sample parents of autistic children, we identified *Ethicality* as a key construct shaping acceptability |
| Minor concerns |  |
| 1. Figure 1 : please input a maximum Y value of 64 for the graph, to help readers integrate the maximum and minimum values of the scale. 2. Please also add a short description of the meaning of the box plot, or clearly state that it is a boxplot in order for readers to find info by themselves to interpret the graph (2 medium quartiles within the box, etc.). 3. Also, what is the meaning of the X? The X of the SLP is not aligned with the median so I don’t understand what the X means. | We have updated the Y axis with a maximum of 64.  A description of the boxplot and the meaning of the X (mean) has been added to the figure. |
| 2. “Data analysis and integration” section (page 10). Just to be completely sure I understood correctly: the authors say that they included data from one parent as soon as they had completed the whole set of questions for one therapy type. They wrote in parentheses that it amounts to 15 questions per intervention type. But in the Appendix A I see 16 questions (15 specific questions and 1 summary question). This 16 is more consistent with the maximum overall score of acceptability (64 = 16 items * 4, if we admit that Strongly Disagree = 0 and Strongly Agree = 4 (except for reverse scoring items). Am I right? If so, maybe change the number in parenthesis line 193 by 16 instead of 15 ? | Thank you for catching this. We have updated line 193 to accurately reflect that 16 questions were included in the creation of the composite score. |
| 3. Table 1, page 15. Again, I love that the authors are so transparent about everything, but here the note « No quotations were from parent respondents who identified as autistic. » felt weird to me as the reader could understand that such answers have been voluntarily removed from Table 1. Since the main text of this section is so nuanced about when a quotation is from an autistic parent or not, maybe this note could be removed from this table? | Agree, we have removed this from the table. |
| 4. Typos : a. Line 136 : alterative should be alternative b. Line 198 : calcualted should be calculated c. Line 199 : socre should be score d. Line 210 : quantiative shoud be quantitative | Thank you. These changes have been corrected. |
