# Supplementary material for: Parent perspectives on services available to autistic children in Canada: A mixed methods survey study
Source: PLoS One. 2026 Aug 20;21(8):e0353678. doi: 10.1371/journal.pone.0353678 (PMC13492781; doi:10.1371/journal.pone.0353678)
Supplement: Supplementary file 7 [file pone.0353678.s008.docx]

May 18 2026

**PLOSOne - Response to Reviewer Comments**

Title: Parent Perspectives on Services Available to Autistic Children in Canada: A Mixed Methods Survey Study

Dear Reviewers,

Thank you for your continued efforts to review this paper. Below are our responses to your suggestions.

| **Reviewer 1** |  |
| --- | --- |
| **Comments** | **Response** |
| The construct of social validity still warrants more attention. The authors could explain why the TFA is a preferable framework (e.g., it is more specific/nuanced), though “social validity lacks rigor, so TFA is better” is not a reasonable argument, as many of the pitfalls discussed in the cited Snodgrass paper could be applied to the use of TFA too (e.g., people creating their own instruments, people defining the constructs as they see fit). To me, these frameworks seem pretty similar, so it would be helpful to know what TFA can give us that social validity can't (or hasn't). | Thank you for probing at this important point. We have further clarified how the TFA aligns with our research question: (lines 113-121):  *The constructs included in this framework provide further breadth than offered by social validity. This framework promotes exploration of how goals and methods align within individual value systems, beyond the extent to which these goals and methods are ‘socially’ appropriate. Exploring the Burden, Opportunity Costs and Perceived Effectiveness is especially relevant to services accessed by autistic children and their families, which can be costly and time intensive.* |
| Thank you to the authors for explaining that they did not explicitly screen for – but also found no evidence of – fraudulent participants. This should be mentioned in the paper itself, as readers will undoubtedly be concerned about fraud, especially since there was a monetary incentive, which always attracts bots/fraudulent folks. Even if there was no systematic screening, it would be helpful to mention that you did not note any incoherent responses. | Thank you for pointing this out – it is important to the reader to understand that we did not have concerns about fraudulent responses.  The following have been added:  *Methods (lines 215-216)*  No measures were included to prevent potential fraudulent responses to the survey (30).  *Results (lines 251-253)*  There was no systemic screening completed for evidence of fraudulent responses; however, there were no incoherent responses nor responses submitted in close succession. |
| REVIEWER 2 |  |
| 1. Page 7, line 135: “Occupations” instead of “occupation”. | We have updated this typo, thank you. |
| 2. Page 15, line 266-271: Among those who identified accessing a therapy, the median acceptability scores were 42 (IQR = 10.25, n = 64) for SLP, 40.5 (IQR = 11.75, n = 74) for OT, and 30 (IQR = 18, n = 35) for ABA. There were no significant differences based on those who had and had not accessed OT and SLP; however, this difference was significant for ABA (t = 3.36, p< 0.01). Thank you for adding this very interesting result. I think it could even be better if such data were included in a graph (maybe boxplots for each therapy type and each group? People who accessed the therapy Vs people who didn’t?). But it’s up to the authors and editorial decision. I don’t want to seem to be pushing for endless rounds of reviews. I just have a minor concern here that needs addressing: the sentence “There were no significant differences based on those who had and had not accessed OT and SLP” is a little bit confusing and could be interpreted as a test of difference between OT and SLP. Maybe rephrase as following: “There were no significant group differences between participants who had access to therapy and those who did not for OT and SLP”. | We have updated the sentence to be clearer. |
| 3. Page 25, line 472-473: “suggesting that parents who did not have personal experience with ABA might have been informed by external sources.” I don’t think this interpretation really adds to the understanding here. I think this result might reflect lingering prejudice against ABA, maybe an heritage of how early punishment procedures used in the 60-70’s by Lovaas or other influential ABA practitioners infused in North American culture (Donvan & Zucker, 2016). Donvan, J., & Zucker, C. (2016). In a different key : The story of autism (p. xiv, 672). Crown Publishers/Random House. | We appreciate this interpretation; however, we are reluctant to extrapolate further on what was informing these perspectives without directly exploring it. |
| 4.Page 29, line 562-563: Maybe “we identified that Ethicality appears to play…” instead of “we identified Ethicality appeared to play”. | Changed, thank you for the suggestion. |
