# Supplementary material for: Parent perspectives on services available to autistic children in Canada: A mixed methods survey study
Source: PLoS One. 2026 Aug 20;21(8):e0353678. doi: 10.1371/journal.pone.0353678 (PMC13492781; doi:10.1371/journal.pone.0353678)
Supplement: Peer review history

# Peer review: Decision Letter 0

**DOI:** 10.1371/journal.pone.0353678.r001

**Authors:** Gökhan Töret

8 Aug 2025

Dear Dr. Caven,

Please submit your revised manuscript by Sep 21 2025 11:59PM. If you will need more time than this to complete your revisions, please reply to this message or contact the journal office at plosone@plos.org. When you're ready to submit your revision, log on to https://www.editorialmanager.com/pone/ and select the 'Submissions Needing Revision' folder to locate your manuscript file.

- A rebuttal letter that responds to each point raised by the academic editor and reviewer(s). You should upload this letter as a separate file labeled 'Response to Reviewers'.
- A marked-up copy of your manuscript that highlights changes made to the original version. You should upload this as a separate file labeled 'Revised Manuscript with Track Changes'.
- An unmarked version of your revised paper without tracked changes. You should upload this as a separate file labeled 'Manuscript'.

If you would like to make changes to your financial disclosure, please include your updated statement in your cover letter. Guidelines for resubmitting your figure files are available below the reviewer comments at the end of this letter.

If applicable, we recommend that you deposit your laboratory protocols in protocols.io to enhance the reproducibility of your results. Protocols.io assigns your protocol its own identifier (DOI) so that it can be cited independently in the future. For instructions see: https://journals.plos.org/plosone/s/submission-guidelines#loc-laboratory-protocols. Additionally, PLOS ONE offers an option for publishing peer-reviewed Lab Protocol articles, which describe protocols hosted on protocols.io. Read more information on sharing protocols at https://plos.org/protocols?utm_medium=editorial-email&utm_source=authorletters&utm_campaign=protocols.

We look forward to receiving your revised manuscript.

Kind regards,

Gökhan Töret

Academic Editor

PLOS ONE

Journal Requirements:

When submitting your revision, we need you to address these additional requirements.

1. Please ensure that your manuscript meets PLOS ONE's style requirements, including those for file naming. The PLOS ONE style templates can be found at

https://journals.plos.org/plosone/s/file?id=wjVg/PLOSOne_formatting_sample_main_body.pdf and

https://journals.plos.org/plosone/s/file?id=ba62/PLOSOne_formatting_sample_title_authors_affiliations.pdf

2. Thank you for stating the following financial disclosure: [This work was supposed by the Kimel Family Graduate Student Scholarship in Pediatric Disability Research and the Hilda and William Courtney Clayton Paediatric Research Fund.].

Please state what role the funders took in the study.  If the funders had no role, please state: """"The funders had no role in study design, data collection and analysis, decision to publish, or preparation of the manuscript.""""

If this statement is not correct you must amend it as needed.

Please include this amended Role of Funder statement in your cover letter; we will change the online submission form on your behalf.

3. Thank you for stating the following in the Competing Interests section:[ M. Penner reports consulting fees from the province of Nova Scotia and Addis & Associates/Roche and research grants from Autism Speaks. The authors have no other disclosures to report].

Please confirm that this does not alter your adherence to all PLOS ONE policies on sharing data and materials, by including the following statement: """"This does not alter our adherence to  PLOS ONE policies on sharing data and materials.” (as detailed online in our guide for authors http://journals.plos.org/plosone/s/competing-interests).  If there are restrictions on sharing of data and/or materials, please state these. Please note that we cannot proceed with consideration of your article until this information has been declared.

Please include your updated Competing Interests statement in your cover letter; we will change the online submission form on your behalf.

4. We note that your Data Availability Statement is currently as follows: [All relevant data are within the manuscript and its Supporting Information files.]

Please confirm at this time whether or not your submission contains all raw data required to replicate the results of your study. Authors must share the “minimal data set” for their submission. PLOS defines the minimal data set to consist of the data required to replicate all study findings reported in the article, as well as related metadata and methods (https://journals.plos.org/plosone/s/data-availability#loc-minimal-data-set-definition).

For example, authors should submit the following data:

- The values behind the means, standard deviations and other measures reported;

- The values used to build graphs;

- The points extracted from images for analysis.

Authors do not need to submit their entire data set if only a portion of the data was used in the reported study.

If your submission does not contain these data, please either upload them as Supporting Information files or deposit them to a stable, public repository and provide us with the relevant URLs, DOIs, or accession numbers. For a list of recommended repositories, please see https://journals.plos.org/plosone/s/recommended-repositories.

If there are ethical or legal restrictions on sharing a de-identified data set, please explain them in detail (e.g., data contain potentially sensitive information, data are owned by a third-party organization, etc.) and who has imposed them (e.g., an ethics committee). Please also provide contact information for a data access committee, ethics committee, or other institutional body to which data requests may be sent. If data are owned by a third party, please indicate how others may request data access.

5. Please include captions for your Supporting Information files at the end of your manuscript, and update any in-text citations to match accordingly. Please see our Supporting Information guidelines for more information: http://journals.plos.org/plosone/s/supporting-information.

6. If the reviewer comments include a recommendation to cite specific previously published works, please review and evaluate these publications to determine whether they are relevant and should be cited. There is no requirement to cite these works unless the editor has indicated otherwise.

[Note: HTML markup is below. Please do not edit.]

Reviewers' comments:

Reviewer's Responses to Questions

**Comments to the Author**

1. Is the manuscript technically sound, and do the data support the conclusions?

Reviewer #1: Yes

Reviewer #2: Partly

**********

2. Has the statistical analysis been performed appropriately and rigorously?

Reviewer #1: Yes

Reviewer #2: Yes

**********

3. Have the authors made all data underlying the findings in their manuscript fully available?

The PLOS Data policy

Reviewer #1: Yes

Reviewer #2: Yes

**********

4. Is the manuscript presented in an intelligible fashion and written in standard English?

Reviewer #1: Yes

Reviewer #2: Yes

**********

Reviewer #1: Parent Perspectives on Services Available to Autistic Children in Canada: A Mixed Methods Survey Study

Thank you for the opportunity to review this important study about parents’ perspectives on services for autistic children in Canada. The authors found varying degrees of support for ABA, OT, and SLP services. Qualitative data suggests that parents have concerns about behavioral approaches and support neurodiversity-affirming practices. This is a very timely paper that will be of interest to many in the field. The study has many strengths, including an advisory group, clear explanation of study methods, and clear reporting of results. Limitations are also clearly identified. Additionally, the paper is well written and easy to follow. Nevertheless, I have several comments related to the method/analysis and have also suggested a variety of literature that could be included to strengthen the rationale for the study and to which the current study’s results could be compared.

Introduction:

-Introduction is very well written and easy to follow. I suggest below a few areas where more detail could be added to make the study rationale stronger.

-The framing of the first part of the introduction could be updated to highlight that the recent research on autistic adults’ perspectives on services is in direct response to autistic people’s voices being ignored for decades. At least in ABA research, when acceptability/satisfaction is assessed, it is usually from the parent’s (or a clinician’s) point of view; rarely is the perspective of the autistic person considered. While it is of course still important to get parents’ perspectives – especially the kind of in-depth perspectives obtained in this study! – there could be more explanation as to why researchers have been focusing more on autistic voices.

-Another larger interview study with autistic adolescents also found several critiques of “social intervention” practices: Bottema-Beutel K, Mullins TS, Harvey MN, Gustafson JR, Carter EW. Avoiding the “brick wall of awkward”: Perspectives of youth with autism spectrum disorder on social-focused intervention practices. Autism. 2016;20(2):196-206. doi:10.1177/1362361315574888

-Another recent survey study looked at autistic and non-autistic individuals’ perspectives on intervention goals. While they did not specifically report on parents, this study may be of interest: Hersh L, Dwyer P, Kapp SK, et al. Community Member Views on Autism Intervention: Effects of Closeness to Autistic People with Intellectual Disabilities And Nonspeaking Autistic People. Autism in Adulthood. 2024;6(3):253-271. doi:10.1089/aut.2023.0202

-Waddington et al. also recently published a qualitative investigation of support goals, which includes input from parents (Waddington H, Jordan P, Scott E, et al. Community perspectives on support goals for young autistic children: A qualitative investigation. Neurodiversity. 2023;1:27546330231218587. doi:10.1177/27546330231218587)

-In ABA, researchers/clinicians are supposed to be assessing “social validity,” which includes appropriateness/acceptability of goals, procedures, and outcomes. While this is certainly done way less than it should be, there are many instances of ABA studies where parents have been asked about their satisfaction with the service provided. However, these assessments are often done in very crude ways, usually without validated instruments. Thus, studies often end up reporting something like, “Parents rated it 6.9/8, so it’s great,” when really we don’t have any way of knowing what “6.9” means, nor do we have nuanced detail about what was good versus bad. You allude to this on page 5, but highlighting this in more detail would strengthen the rationale for your study, because your study actually goes into much more nuance.

-Even if social validity assessment in ABA has been a bit of a mess, many researchers likely see social validity as a theoretical framework that incorporates the significance of goals, appropriateness of procedures, and importance of the effects (Wolf, 1978; for an updated framework, see: Snodgrass MR, Chung MY, Kretzer JM, Biggs EE. Rigorous Assessment of Social Validity: A Scoping Review of a 40-Year Conversation. Remedial and Special Education. 2022;43(2):114-130. doi:10.1177/07419325211017295). Therefore, the authors should reconsider saying that no existing research has explored parental perspectives through the lens of a theoretical framework.

-The introduction would also benefit from more explanation of what ABA, OT, and SLP services look like.

Methods:

-The PAG is a huge strength of this study! Also great explanation as to how an advisory board differs from true CBPR.

-On page 8, consider saying no “published” or “validated” survey relating to acceptability of autism services exists, as multiple other studies have used questionnaires to ask about this (eg the one used by Waddington, the “Autism Intervention Attitudes Scale” used by Hersh et al. and Baiden et al.).

-Were there any inclusion/exclusion criteria related to age of child?

-Were any measures taken to ensure data integrity (i.e., to ensure participants were “real” and not fraudulent?).

Results:

-Were parents asked about whether children were currently receiving the service and, if not, how long ago their children were enrolled? (Sorry, I couldn’t find the demographics survey in the supplemental materials.) This could impact acceptability ratings.

-Table 1 makes it seem like OT was universally associated with neurodiversity-affirming practices, SLP was associated with potential harms, etc. While I can tell from the narrative that this is not the case, this is somewhat confusing.

-It should be made clearer that parents were asked about services even if their child had never received such services. It would also be helpful, if possible, to look into whether responses differed significantly between those whose children had versus had not participated in the service (especially for ABA since it was about 50/50). A critique of research with autistic adults is that some never had ABA, so those folks’ perspectives are not relevant. (To be clear, I do not agree with this, but it is a critique I’ve seen.) Some might have this critique of parents in this study who are commenting on ABA but whose children have never engaged in it. If not possible to look into this more, this could just be discussed more the discussion.

Discussion:

-The authors may also want to consider citing a recent qualitative study of autistic adults’ perspectives on “intervention” goals, as many of these studies are showing that parents’ and autistic adults’ perspectives converge: Schuck RK, Geng A, Doss Y, et al. A qualitative investigation into autistic adults’ perspectives on intervention goals for autistic children. Neurodiversity. 2024;2:27546330241266718. doi:10.1177/27546330241266718

-The authors make a great point about behavioral approaches being present across services!

-See another recent study about parents’ views of neurodiversity: Dwyer P, Gurba AN, Kapp SK, et al. Community views of neurodiversity, models of disability and autism intervention: Mixed methods reveal shared goals and key tensions. Autism. Published online September 18, 2024:13623613241273029. doi:10.1177/13623613241273029

Reviewer #2: In this very interesting article, the authors designed a survey to assess how parents of autistic individuals perceive three types of intervention (Occupational Therapy (OT), Speech Language Pathology (SLP) and Applied Behaviour Analysis (ABA)), using an acceptability framework and a mix of quantitative and qualitative methods. They found that the overall acceptability of OT and SLP (overall score of 40-42 out of 64) was higher than ABA (overall score of 28). They also elaborate on the verbal responses of participants to identify key components affecting how they perceive the acceptability of these methods. I found the article well written. I really appreciate the high level of transparency that the authors put into their work, and also the level of care displayed for all the groups involved in the study (autistic children and adults, parents, clinicians, students). I do have several major and minor points that need to be addressed. In particular, I think that additional analyses could be performed on the quantitative data.

Major concerns

1. Why focus on these three interventions and not others? Have they been chosen because they are one on one interventions and not group ones? Some very important types of interventions seem to have been left out of this work (e.g. parent training, early sociodevelopmental interventions such as the Denver Model, etc.). Please provide a rationale for focusing on these three interventions only. Please also provide summary descriptions of these three types of intervention.

2. One possible bias affecting the results is that parents whose child did not benefit from the intervention could also provide answers regarding that type of intervention. Are the ABA related results credible, since only half of the parents had the occasion to engage with this form of intervention? Are the quantitative results different between the participants familiar with ABA (i.e. with their child having benefited from ABA) and the others?

3. I would recommend providing a scatterplot of participants by age, just to get a full picture of the age demography of the sample. Also, it could have been interesting to make the parents assess the approximate date of intervention for their child(ren), to get a grasp of a possible effect of date/time on their responses (maybe more recent interventions are more acceptable ? Regardless of intervention type?). Please provide some additional data if you have some, or maybe just comment on this point in the limitation section? Maybe you could get this info if you cross sociodemographical data from the parents (their age at moment of survey for example) with the « age beginning therapy » variable available in Table 1. Of course this suggestion might be too complex for the qualitative data, but maybe it could be worthy to try it on quantitative data?

4. Some of the durations of therapy seem to be really extreme (up to 16 years for SLP and OT). Are these profiles associated with peculiar answers or attitudes toward the interventions? The median value is also very interesting, since all intervention types have a median duration of 2 to 3 years, which is more than what is usually recommended or allowed by public fundings (but I come from a French background).

5. Page 27, last paragraph of the conclusion. « In conclusion, across this sample parents of autistic children, Ethicality was a key construct shaping acceptability ». It is an interesting view but nothing in the design or the results allow for prioritizing this dimension over Effectiveness and Self-Efficacy. One methodology that could have solved this is to make participants rank the 7 TFA dimensions from most to least important to them, but I don’t think the authors used this kind of methodology?

Minor concerns

1. Figure 1 : please input a maximum Y value of 64 for the graph, to help readers integrate the maximum and minimum values of the scale. Please also add a short description of the meaning of the box plot, or clearly state that it is a boxplot in order for readers to find info by themselves to interpret the graph (2 medium quartiles within the box, etc.). Also, what is the meaning of the X? The X of the SLP is not aligned with the median so I don’t understand what the X means.

2. “Data analysis and integration” section (page 10). Just to be completely sure I understood correctly: the authors say that they included data from one parent as soon as they had completed the whole set of questions for one therapy type. They wrote in parentheses that it amounts to 15 questions per intervention type. But in the Appendix A I see 16 questions (15 specific questions and 1 summary question). This 16 is more consistent with the maximum overall score of acceptability (64 = 16 items * 4, if we admit that Strongly Disagree = 0 and Strongly Agree = 4 (except for reverse scoring items). Am I right? If so, maybe change the number in parenthesis line 193 by 16 instead of 15 ?

3. Table 1, page 15. Again, I love that the authors are so transparent about everything, but here the note « No quotations were from parent respondents who identified as autistic. » felt weird to me as the reader could understand that such answers have been voluntarily removed from Table 1. Since the main text of this section is so nuanced about when a quotation is from an autistic parent or not, maybe this note could be removed from this table?

4. Typos :

a. Line 136 : alterative should be alternative

b. Line 198 : calcualted should be calculated

c. Line 199 : socre should be score

d. Line 210 : quantiative shoud be quantitative

**********

what does this mean?). If published, this will include your full peer review and any attached files.

If you choose “no”, your identity will remain anonymous but your review may still be made public.

**Do you want your identity to be public for this peer review?**  For information about this choice, including consent withdrawal, please see our Privacy Policy

Reviewer #1: No

Reviewer #2: **Yes:** Matias Baltazar

**********

[NOTE: If reviewer comments were submitted as an attachment file, they will be attached to this email and accessible via the submission site. Please log into your account, locate the manuscript record, and check for the action link "View Attachments". If this link does not appear, there are no attachment files.]

While revising your submission, please upload your figure files to the Preflight Analysis and Conversion Engine (PACE) digital diagnostic tool, https://pacev2.apexcovantage.com/. PACE helps ensure that figures meet PLOS requirements. To use PACE, you must first register as a user. Registration is free. Then, login and navigate to the UPLOAD tab, where you will find detailed instructions on how to use the tool. If you encounter any issues or have any questions when using PACE, please email PLOS at figures@plos.org. Please note that Supporting Information files do not need this step.

# Author comment: Author response to Decision Letter 1

**DOI:** 10.1371/journal.pone.0353678.r002

4 Mar 2026

We thank the reviewers for their time and valuable feedback. We have included an itemized response document.

Attachment Submitted filename: PLOS ONE Reviewer Responses_Final.docx

# Peer review: Decision Letter 1

**DOI:** 10.1371/journal.pone.0353678.r003

**Authors:** Gökhan Töret

14 Apr 2026

PONE-D-25-32240R1

Parent Perspectives on Services Available to Autistic Children in Canada: A Mixed Methods Survey Study

PLOS One

Dear Dr. Caven,

Thank you for submitting your manuscript to PLOS ONE. After careful consideration, we feel that it has merit but does not fully meet PLOS ONE’s publication criteria as it currently stands. Therefore, we invite you to submit a revised version of the manuscript that addresses the points raised during the review process.

Please submit your revised manuscript by May 29 2026 11:59PM. If you will need more time than this to complete your revisions, please reply to this message or contact the journal office at plosone@plos.org. When you're ready to submit your revision, log on to https://www.editorialmanager.com/pone/ and select the 'Submissions Needing Revision' folder to locate your manuscript file.

Please include the following items when submitting your revised manuscript:

- A letter that responds to each point raised by the academic editor and reviewer(s). You should upload this letter as a separate file labeled 'Response to Reviewers'.
- A marked-up copy of your manuscript that highlights changes made to the original version. You should upload this as a separate file labeled 'Revised Manuscript with Track Changes'.
- An unmarked version of your revised paper without tracked changes. You should upload this as a separate file labeled 'Manuscript'.

If you would like to make changes to your financial disclosure, please include your updated statement in your cover letter. Guidelines for resubmitting your figure files are available below the reviewer comments at the end of this letter.

If applicable, we recommend that you deposit your laboratory protocols in protocols.io to enhance the reproducibility of your results. Protocols.io assigns your protocol its own identifier (DOI) so that it can be cited independently in the future. For instructions see: https://journals.plos.org/plosone/s/submission-guidelines#loc-laboratory-protocols. Additionally, PLOS ONE offers an option for publishing peer-reviewed Lab Protocol articles, which describe protocols hosted on protocols.io. Read more information on sharing protocols at https://plos.org/protocols?utm_medium=editorial-email&utm_source=authorletters&utm_campaign=protocols.

As the corresponding author, your ORCID iD is verified in the submission system and will appear in the published article. PLOS supports the use of ORCID, and we encourage all coauthors to register for an ORCID iD and use it as well. Please encourage your coauthors to verify their ORCID iD within the submission system before final acceptance, as unverified ORCID iDs will not appear in the published article. *Only*  the individual author can complete the verification step; PLOS staff *cannot*  verify ORCID iDs on behalf of authors.

We look forward to receiving your revised manuscript.

Kind regards,

Gökhan Töret

Academic Editor

PLOS One

**Journal Requirements:**

1. If the reviewer comments include a recommendation to cite specific previously published works, please review and evaluate these publications to determine whether they are relevant and should be cited. There is no requirement to cite these works unless the editor has indicated otherwise.

2. Please review your reference list to ensure that it is complete and correct. If you have cited papers that have been retracted, please include the rationale for doing so in the manuscript text, or remove these references and replace them with relevant current references. Any changes to the reference list should be mentioned in the rebuttal letter that accompanies your revised manuscript. If you need to cite a retracted article, indicate the article’s retracted status in the References list and also include a citation and full reference for the retraction notice.

[Note: HTML markup is below. Please do not edit.]

Reviewers' comments:

Reviewer's Responses to Questions

**Comments to the Author**

1. If the authors have adequately addressed your comments raised in a previous round of review and you feel that this manuscript is now acceptable for publication, you may indicate that here to bypass the “Comments to the Author” section, enter your conflict of interest statement in the “Confidential to Editor” section, and submit your "Accept" recommendation.

Reviewer #1: (No Response)

Reviewer #2: All comments have been addressed

**********

2. Is the manuscript technically sound, and do the data support the conclusions?

The manuscript must describe a technically sound piece of scientific research with data that supports the conclusions. Experiments must have been conducted rigorously, with appropriate controls, replication, and sample sizes. The conclusions must be drawn appropriately based on the data presented.

Reviewer #1: Yes

Reviewer #2: Yes

**********

3. Has the statistical analysis been performed appropriately and rigorously?

Reviewer #1: Yes

Reviewer #2: Yes

**********

4. Have the authors made all data underlying the findings in their manuscript fully available?

The PLOS Data policy requires authors to make all data underlying the findings described in their manuscript fully available without restriction, with rare exception (please refer to the Data Availability Statement in the manuscript PDF file). The data should be provided as part of the manuscript or its supporting information, or deposited to a public repository. For example, in addition to summary statistics, the data points behind means, medians and variance measures should be available. If there are restrictions on publicly sharing data—e.g. participant privacy or use of data from a third party—those must be specified.

Reviewer #1: No

Reviewer #2: No

**********

5. Is the manuscript presented in an intelligible fashion and written in standard English?

PLOS ONE does not copyedit accepted manuscripts, so the language in submitted articles must be clear, correct, and unambiguous. Any typographical or grammatical errors should be corrected at revision, so please note any specific errors here.

Reviewer #1: Yes

Reviewer #2: Yes

**********

6. Review Comments to the Author

Please use the space provided to explain your answers to the questions above. You may also include additional comments for the author, including concerns about dual publication, research ethics, or publication ethics. (Please upload your review as an attachment if it exceeds 20,000 characters)

Reviewer #1: Thank you for the opportunity to review the revised version of "Parent Perspectives on Services Available to Autistic Children in Canada: A Mixed Methods Survey Study." The authors were very responsive to the reviewers' comments, and their revisions greatly strengthen the paper. I just have two comments that I feel need to be addressed before endorsing publication:

1. The construct of social validity still warrants more attention. The authors could explain why the TFA is a preferable framework (e.g., it is more specific/nuanced), though “social validity lacks rigor, so TFA is better” is not a reasonable argument, as many of the pitfalls discussed in the cited Snodgrass paper could be applied to the use of TFA too (e.g., people creating their own instruments, people defining the constructs as they see fit). To me, these frameworks seem pretty similar, so it would be helpful to know what TFA can give us that social validity can't (or hasn't).

2. Thank you to the authors for explaining that they did not explicitly screen for – but also found no evidence of – fraudulent participants. This should be mentioned in the paper itself, as readers will undoubtedly be concerned about fraud, especially since there was a monetary incentive, which always attracts bots/fraudulent folks. Even if there was no systematic screening, it would be helpful to mention that you did not note any incoherent responses.

Reviewer #2: I thank the authors for their hard work addressing my concerns quite successfully. I just have a few minor concerns left (I used the track changes version for my second round of review, when referencing pages and lines):

1. Page 7, line 135: “Occupations” instead of “occupation”.

2. Page 15, line 266-271: Among those who identified accessing a therapy, the median acceptability scores were 42 (IQR = 10.25, n = 64) for SLP, 40.5 (IQR = 11.75, n = 74) for OT, and 30 (IQR = 18, n = 35) for ABA. There were no significant differences based on those who had and had not accessed OT and SLP; however, this difference was significant for ABA (t = 3.36, p< 0.01).

Thank you for adding this very interesting result. I think it could even be better if such data were included in a graph (maybe boxplots for each therapy type and each group? People who accessed the therapy Vs people who didn’t?). But it’s up to the authors and editorial decision. I don’t want to seem to be pushing for endless rounds of reviews.

I just have a minor concern here that needs addressing: the sentence “There were no significant differences based on those who had and had not accessed OT and SLP” is a little bit confusing and could be interpreted as a test of difference between OT and SLP. Maybe rephrase as following: “There were no significant group differences between participants who had access to therapy and those who did not for OT and SLP”.

3. Page 25, line 472-473: “suggesting that parents who did not have personal experience with ABA might have been informed by external sources.”

I don’t think this interpretation really adds to the understanding here. I think this result might reflect lingering prejudice against ABA, maybe an heritage of how early punishment procedures used in the 60-70’s by Lovaas or other influential ABA practitioners infused in North American culture (Donvan & Zucker, 2016).

Donvan, J., & Zucker, C. (2016). In a different key : The story of autism (p. xiv, 672). Crown Publishers/Random House.

4. Page 29, line 562-563: Maybe “we identified that Ethicality appears to play…” instead of “we identified Ethicality appeared to play”.

**********

7. PLOS authors have the option to publish the peer review history of their article (what does this mean?). If published, this will include your full peer review and any attached files.

If you choose “no”, your identity will remain anonymous but your review may still be made public.

**Do you want your identity to be public for this peer review?**  For information about this choice, including consent withdrawal, please see our Privacy Policy.

Reviewer #1: No

Reviewer #2: **Yes:** Matias Baltazar

**********

[NOTE: If reviewer comments were submitted as an attachment file, they will be attached to this email and accessible via the submission site. Please log into your account, locate the manuscript record, and check for the action link "View Attachments". If this link does not appear, there are no attachment files.]

To ensure your figures meet our technical requirements, please review our figure guidelines: https://journals.plos.org/plosone/s/figures

You may also use PLOS’s free figure tool, NAAS, to help you prepare publication quality figures: https://journals.plos.org/plosone/s/figures#loc-tools-for-figure-preparation.

NAAS will assess whether your figures meet our technical requirements by comparing each figure against our figure specifications.

# Author comment: Author response to Decision Letter 2

**DOI:** 10.1371/journal.pone.0353678.r004

12 Jun 2026

See attached document for responses to reviewers.

Attachment Submitted filename: Responses to Reviewers PLOS One May 2026.docx

# Peer review: Decision Letter 2

**DOI:** 10.1371/journal.pone.0353678.r005

**Authors:** Gökhan Töret

29 Jun 2026

Parent Perspectives on Services Available to Autistic Children in Canada: A Mixed Methods Survey Study

PONE-D-25-32240R2

Dear Dr. Caven,

We’re pleased to inform you that your manuscript has been judged scientifically suitable for publication and will be formally accepted for publication once it meets all outstanding technical requirements.

Within one week, you’ll receive an e-mail detailing the required amendments. When these have been addressed, you’ll receive a formal acceptance letter and your manuscript will be scheduled for publication.

An invoice will be generated when your article is formally accepted. Please note, if your institution has a publishing partnership with PLOS and your article meets the relevant criteria, all or part of your publication costs will be covered. Please make sure your user information is up-to-date by logging into Editorial Manager at Editorial Manager® and clicking the ‘Update My Information' link at the top of the page. For questions related to billing, please contact billing support.

If your institution or institutions have a press office, please notify them about your upcoming paper to help maximize its impact. If they’ll be preparing press materials, please inform our press team as soon as possible -- no later than 48 hours after receiving the formal acceptance. Your manuscript will remain under strict press embargo until 2 pm Eastern Time on the date of publication. For more information, please contact onepress@plos.org.

Kind regards,

Gökhan Töret

Academic Editor

PLOS One

Additional Editor Comments (optional):

Reviewers' comments:

Reviewer's Responses to Questions

**Comments to the Author**

Reviewer #1: All comments have been addressed

Reviewer #2: All comments have been addressed

**********

2. Is the manuscript technically sound, and do the data support the conclusions?

Reviewer #1: Yes

Reviewer #2: Yes

**********

3. Has the statistical analysis been performed appropriately and rigorously?

Reviewer #1: Yes

Reviewer #2: Yes

**********

4. Have the authors made all data underlying the findings in their manuscript fully available?

The PLOS Data policy

Reviewer #1: No

Reviewer #2: No

**********

5. Is the manuscript presented in an intelligible fashion and written in standard English?

Reviewer #1: Yes

Reviewer #2: Yes

**********

Reviewer #1: Thank you again for the opportunity to review this paper. The authors have been very responsive to the reviewers' comments. The paper is ready for publication in my opinion.

Reviewer #2: (No Response)

**********

what does this mean?). If published, this will include your full peer review and any attached files.

If you choose “no”, your identity will remain anonymous but your review may still be made public.

**Do you want your identity to be public for this peer review?**  For information about this choice, including consent withdrawal, please see our Privacy Policy

Reviewer #1: No

Reviewer #2: **Yes:** Matias Baltazar

**********

# Editor report: Acceptance letter

**DOI:** 10.1371/journal.pone.0353678.r006

**Authors:** Gökhan Töret

PONE-D-25-32240R2

PLOS One

Dear Dr. Caven,

I'm pleased to inform you that your manuscript has been deemed suitable for publication in PLOS One. Congratulations! Your manuscript is now being handed over to our production team.

At this stage, our production department will prepare your paper for publication. This includes ensuring the following:

* All references, tables, and figures are properly cited

* All relevant supporting information is included in the manuscript submission,

* There are no issues that prevent the paper from being properly typeset

You will receive further instructions from the production team, including instructions on how to review your proof when it is ready. Please keep in mind that we are working through a large volume of accepted articles, so please give us a few days to review your paper and let you know the next and final steps.

Lastly, if your institution or institutions have a press office, please let them know about your upcoming paper now to help maximize its impact. If they'll be preparing press materials, please inform our press team within the next 48 hours. Your manuscript will remain under strict press embargo until 2 pm Eastern Time on the date of publication. For more information, please contact onepress@plos.org.

You will receive an invoice from PLOS for your publication fee after your manuscript has reached the completed accept phase. If you receive an email requesting payment before acceptance or for any other service, this may be a phishing scheme. Learn how to identify phishing emails and protect your accounts at https://explore.plos.org/phishing.

If we can help with anything else, please email us at customercare@plos.org.

Thank you for submitting your work to PLOS ONE and supporting open access.

Kind regards,

PLOS ONE Editorial Office Staff

on behalf of

Dr. Gökhan Töret

Academic Editor

PLOS One
